# Dietary Influences on Nitrogen and Phosphorus Footprints in Indian Food Systems: A State and Union Territory-Level Analysis

**DOI:** 10.3390/nu17233758

**Published:** 2025-11-29

**Authors:** Aurup Ratan Dhar, Azusa Oita, Himadri Kaushik, Ananta Narayan Panda, Tapan Kumar Adhya, Kazuyo Matsubae

**Affiliations:** 1 Research Institute for Humanity and Nature, 457-4 Kamigamo Motoyama, Kita-ku, Kyoto 603-8047, Japan; aurup971@gmail.com (A.R.D.); 2 Graduate School of Environmental Studies, Tohoku University, 468-1 Aoba, Aramaki, Aoba-ku, Sendai 980-0845, Japan; kazuyo.matsubae.a2@tohoku.ac.jp (K.M.); 3 Institute for Agro-Environmental Sciences, National Agriculture and Food Research Organization, 3-1-3, Kannondai, Tsukuba 305-8604, Japan; 4 School of Biotechnology, Kalinga Institute of Industrial Technology (Deemed University), Bhubaneswar 751024, India; himadrikaushik18@gmail.com (H.K.); anpandabiotek@gmail.com (A.N.P.)

**Keywords:** bottom-up approach, dietary choice, food consumption, food waste, nutrient management, nutrient use efficiency

## Abstract

**Background/Objectives:** Nitrogen (N) and phosphorus (P) are essential macronutrients for crop production. However, their losses throughout the agri-food system pose significant environmental and public health risks. India, with its diverse dietary cultures and large agricultural sector, presents a unique context for evaluating nutrient footprints. This study aims to provide the first sub-national assessment of food-related N and P footprints across Indian states and union territories, evaluating how vegetarian and non-vegetarian diets influence these footprints. **Methods:** This study employed a diet-sensitive bottom-up approach using national dietary consumption statistics from 2011–2012 to estimate food N and P footprints. The analysis incorporated regional dietary profiles and nutrient use efficiencies in crop production, along with food waste data, to quantify the affecting factors. **Results:** The national average food footprints were estimated at 13.11 kg-N capita^−1^ year^−1^ and 1.16 kg-P capita^−1^ year^−1^, with sub-national variation ranging from 52% to 144% of the national average for N, and 46% to 166% for P. Regions with prevalent non-vegetarian diets exhibited significantly higher footprints than those with vegetarian diets. Low nutrient use efficiencies (NUE 19%, PUE 31%) and consumer-level food waste (contributing nearly 4%) were also identified as key drivers of elevated footprints. **Conclusions:** The findings indicate that dietary choices, agricultural nutrient management, and food waste practices collectively contribute to the nutrient-related risks in India. Enhancing nutrient use efficiency, promoting plant-based diets, and improving waste management in culturally and regionally sensitive ways are crucial for reducing N and P losses. These findings provide actionable insights for the development of sustainable nutrition and agro-environmental policies.

## 1. Introduction

Nitrogen (N) and phosphorus (P) are macronutrients, essential for all living organisms and key components of a wide range of fertilizers used for agricultural production [1]. Since 1960s, N and P fertilizer use has witnessed an upward surge [2] and their inputs into food production substantially exceed their presence in consumed foods [3]. Losses of N and P along processes of the food system (i.e., cultivation, harvesting, fattening, transportation, processing, packaging, distribution, consumption, and disposal of residuals) result in excess of N and P in reactive forms in air and water. This has created a global concern from viewpoints of adverse environmental impacts, ecosystem vulnerability, biodiversity loss, and human health issues [4]. Nitrous oxide (N_2_O) emissions, particularly from the production process, play a significant role, with consequences such as acid rain and ozone layer depletion. These changes also pose human health risks including respiratory problems and skin cancer [5]. In addition, the increasing demand for P in the food system poses environmental challenges, including nutrient enrichment and eutrophication of water bodies [6,7,8]. Therefore, mitigating these nutrient losses has become an urgent priority to protect ecosystem health and human well-being [1].

Over the recent years, quantifying N and P resource use with lifecycle aspects has been shown to be a powerful approach towards sustainable N and P management [3,9,10,11,12,13,14,15,16,17,18]. Among them, the N footprint quantifies the amount of reactive nitrogen (N*r*, nitrogen compounds other than N_2_ gas) released to the environment due to our food production and consumption as well as non-food production and energy use [9]. The P footprint is concerned with the amount of P resource (i.e., mined P) required to produce the food consumed [10,14,15,16,17,18]. To integrally tackle the N and P issues, a common framework to calculate the food N and P footprint using the *N*- and *P*-Calculator method was proposed by Oita et al. (2020) [3]. A thorough understanding of these N and P footprints is critical to increasing consumer awareness of the environmental impacts of their food choices, thus bridging the gap between individual behavior and global environmental concerns [19].

Global studies show large geographic variation in per capita food N and P footprints and clear links to dietary composition [10,20]. Temporal trends further indicate that dietary transitions toward more animal-based foods in emerging economies have sharply increased N and P footprints over recent decades [3]. In addition to cross-country differences, sub-national assessments reveal substantial heterogeneity within countries. For example, in China, urbanization and rising incomes have elevated food N footprints in cities, while rural areas suffer higher nutrient losses due to poor waste treatment infrastructure [21]. McCourt and MacDonald (2021) conducted a sub-national assessment in Canada [22], uncovering notable provincial disparities in N footprints and emphasizing the value of localized data for a more comprehensive understanding of nutrient footprints. India, the largest country in South Asia by area and population, plays a central role in the global agri-food system. Emissions from the food system contribute significantly to India’s total greenhouse gas (GHG) emissions, with synthetic fertilizers being a major contributor [23]. Following the common framework by Oita et al. (2020) and the modified calculation for religion-specific food N and P footprints by Dhar et al. (2021) [3,19], Dhar (2022) estimated the food N and P footprints of the countries in the Indian subcontinent region taking into consideration cultural and religious aspects of diet [24]. The authors found that the food N and P footprints of the population in the Indian subcontinent actually represent a spectrum among religious communities—lower footprints in largely plant-based diets and higher footprints with more animal-sourced foods. Furthermore, India’s major grain-producing “food bowl” states are left with excessive nutrient surpluses due to interstate food trade, contributing to polluted waterways and soil nutrient imbalances in those regions [25].

These prior findings highlight that diet composition and nutrient management practices are critical determinants of N and P footprints, and they reinforce the need for studies at finer scales. However, N and P footprints at the sub-national level in India are still undetermined, as no prior study has assessed nutrient footprints at the state or union-territory level within India, which represents a critical knowledge gap. The unique religious and cultural diversity across India’s 28 states and 7 union territories [26] (Figure S1a,b) contributes to significant variations in food consumption patterns. This diversity underscores the importance of analyzing N and P footprints at a finer sub-national resolution, since national averages may conceal significant regional disparities. The national-level assessments on food N and P footprints in the previous studies often relied on datasets that overlooked regional variations in dietary habits, agricultural practices, socioeconomic factors, and environmental conditions within a country. This study addresses this gap by conducting a case study in India, a nation characterized by pronounced regional diversity. By utilizing state- and union territory-level data from Indian government sources, this study provides fine-scale analysis of spatial variations in N and P footprints across the country. This localized approach enhances the understanding of N and P footprints within diverse regional contexts. Furthermore, it offers insights for region-specific policy interventions aimed at sustainable N and P management and mitigate environmental impacts.

Dietary choices in India are profoundly influenced by its rich religious and cultural diversity, as the population practices a diverse array of faiths with 79.8% of the population being Hindu, 14.2% Muslim, 2.3% Christian, 1.7% Sikh, 0.8% Buddhist, 0.4% Jain, and 1.2% other [27]. At the sub-national levels, although most of the population of states and union territories follow Hinduism, the majority of the population of Lakshadweep and “Jammu and Kashmir” follow Islam. Mizoram, Nagaland, Meghalaya, Manipur, and Arunachal Pradesh, on the contrary, are primarily populated by followers of Christianity, and the people of Punjab are primarily followers of Sikhism [28]. This religious diversity is reflected in variations in food consumption patterns, particularly in terms of vegetarianism and non-vegetarianism [29]. These differences present an opportunity to investigate how diet influences N and P footprints and associated environmental impacts.

The primary objective of this study is to examine how dietary preferences—particularly the prevalence of vegetarian versus non-vegetarian diets—affects the N and P footprints of India’s states and union territories. To accomplish this, we adapted existing *N*- and *P*- calculators and customized them for vegetarian and non-vegetarian diets—an important distinction that reflects India’s diverse religious practices. This adapted approach allows for a more culturally nuanced and precise analysis at the sub-national level. It is presumed that states and union territories with predominantly non-vegetarian diets will exhibit higher per capita food N and P footprints than those with mostly vegetarian diets, due to the greater nutrient losses associated with animal-based food production. The goal of this study is to unravel the complex interplay between dietary habits, religious diversity, and their environmental impacts, with the goal of providing insights that could guide the development of targeted N and P management policies that resonate with India’s rich cultural tapestry. The findings of this study will contribute significantly to local and national environmental policy-making in India. More broadly, they will serve to illustrate the extent and insights of diversity within a large country with multicultural and multi-geographical contexts. Such findings will be of great value in global discourse on sustainable agricultural practices and citizen environmental action.

The remainder of this paper is organized as follows: Section 2 describes the materials and methods used, including data sources and the diet-specific footprint calculation approach. Section 3 presents the results of the state-level N and P footprint analysis, while Section 4 discusses the key patterns and drivers observed, and implications for policy. Finally, Section 5 outlines the conclusions and future research based on this study’s findings.

## 2. Materials and Methods

**Figure 1:**
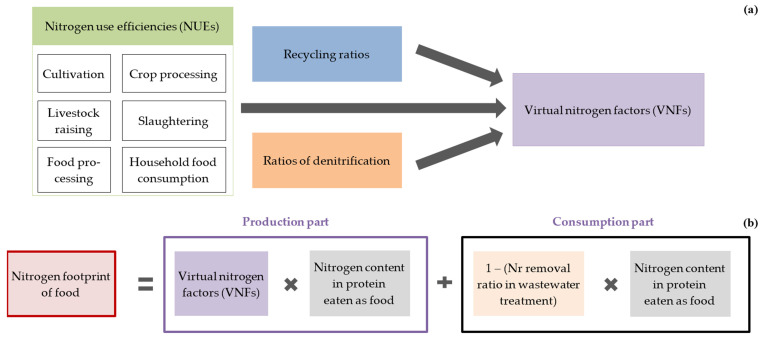
Relationships between nitrogen use efficiencies (NUEs) and virtual nitrogen factors (VNFs) in the calculation of the nitrogen footprint of food: (**a**) VNFs are integrated factors derived from NUEs of four/six steps in crop/meat production; (**b**) the production component of the nitrogen footprint of food is computed by multiplying VNFs by the nitrogen content in protein consumed as food. Adding the consumption component yields the total nitrogen footprint of food. Similarly, virtual phosphorus factors (VPFs), calculated from phosphorus use efficiencies (PUEs), are multiplied by the phosphorus content in food intake to determine the production component of the phosphorus footprint of food.

The N or P footprints of food at the state and union territory-levels were calculated based on the nutrient content of consumed food. This was done by multiplying corresponding food item-specific factors representing nutrient losses per unit intake—namely, virtual N factors (VNFs) and virtual P factors (VPFs) for production—and factors for Nr removal in wastewater treatment (Figure 1a,b). This study adopted the religion-sensitive *N*- and *P*-Calculator methods developed by Dhar et al. (2021) [19], incorporating the dietary patterns of vegetarians and non-vegetarians were considered for the calculation. These methods estimate N and P losses across the food supply chain using dietary intake, nutrient composition of food items, food waste ratios, and production-stage loss factors. For each dietary group, the per capita food N and P footprints are calculated by combining food intake, nutrient content, the consumer level waste proportions, and loss factors during production and processing. The details of these diet-specific *N*- and *P*-Calculator methods are as follows.

### 2.1. Diet Patterns Considered and Data Sources

To calculate the N and P footprints of food, it is necessary to know the amount of each food item consumed. Since the consumption of many food items in India is subject to the country’s rules and regulations, it is assumed that the consumption of individual food items differs significantly between vegetarians and non-vegetarians. Most followers of the Jainism, Hinduism, Sikhism, and Buddhism are strict vegetarians. Christians have dietary restrictions, while Muslims consume non-vegetarian food but follow *halal* slaughter practices and avoid pork [30]. On an average in 2011–2012, vegetarians accounted for 25.8% of the Indian population, while non-vegetarians accounted for 74.2%. Although the overall proportion of vegetarians at the national level is close to a quarter, the proportion was greater than half in the following states and union territories: Rajasthan, Haryana, Punjab, Gujarat, Himachal Pradesh, Madhya Pradesh, Chandigarh, D. & N. Haveli, and Daman & Diu (Figure S1b; [28]).

Dietary regulations for vegetarians and non-vegetarians were considered to categorize the dietary habits. The vegetarian diet is defined to allow only plant-based foods, excluding all animal-based foods except milk and dairy products. There are no category-wide food restrictions for the non-vegetarian diet [31,32]. Data for commonly consumed food items at the state and union territory levels were obtained from the websites of the Ministry of Program Implementation and Statistics (https://www.mospi.gov.in/, accessed on 21 October 2022) and the Ministry of Agriculture and Farmers Welfare (https://www.agriwelfare.gov.in/, accessed on 21 October 2022), categorized according to N and P intake by each dietary group (2011–2012).

### 2.2. Food Nitrogen and Phosphorus Footprints

**Figure 2:**
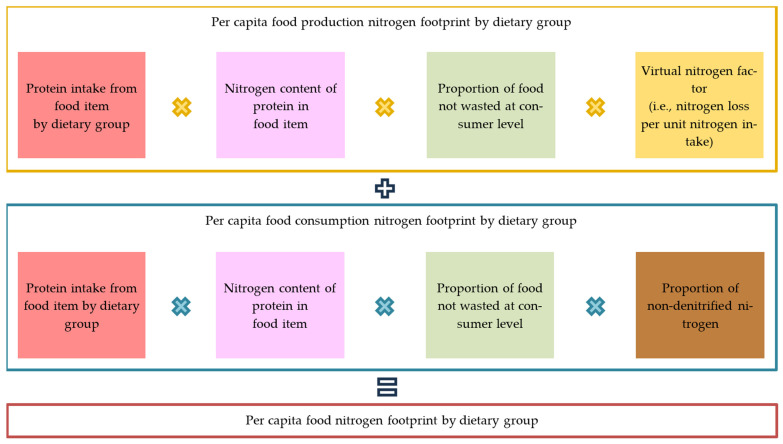
Calculation schematic for the food nitrogen footprint of a dietary group.

The per capita food nitrogen footprint of a dietary group is the sum of production and consumption footprints. It is calculated using the protein contained in the food supplied, the nitrogen content per protein unit, consumer-level waste proportion, and factors for reactive nitrogen loss during production or non-denitrified nitrogen. (Figure 2). Similarly, the per capita food phosphorus footprint of a dietary group was calculated using food consumption amount, phosphorus content, consumer-level waste proportion, and production-stage loss factors. The per capita food N or P footprints of states and union territories were calculated as the weighted average of the per capita food N or P footprints of the vegetarian and non-vegetarian groups in each area.

In the *N*-Calculator method, production and consumption parts are calculated from protein content of the food supplied (Prot_Sup), multiplied by N content (Prot_Ncont), consumer-level waste proportion (1 − FdWst), and factors for Nr loss during production (VNF_Dom) or removal in wastewater treatment (RDenit). The following equations were used to estimate the per capita food N footprint (FdNF) of the state/union territory *ɵ* as the sum of the production part (FdNF_Prod) (Equation (1)) and the consumption part (FdNF_Cons) (Equation (2)), in a weighted average of the vegetarian and non-vegetarian groups (Equation (3)):

(1) $${\mathrm{FdNF}\_\mathrm{Prod}}_{\mathrm{hk}ɵ}\;=\;{\mathrm{Prot}\_\mathrm{Sup}}_{\mathrm{hk}ɵ}\;\times \;{\mathrm{Prot}\_\mathrm{Ncont}}_{h}\;\times \;\left(1-{\mathrm{FdWst}}_{hɵ}\right)\;\times \;{\mathrm{VNF}\_\mathrm{Dom}}_{hɵ},$$

(2) $${\mathrm{FdNF}\_\mathrm{Cons}}_{\mathrm{hk}ɵ}={\mathrm{Prot}\_\mathrm{Sup}}_{\mathrm{hk}ɵ}\;\times \;{\mathrm{Prot}\_\mathrm{Ncont}}_{h}\;\times \;\left(1-{\mathrm{FdWst}}_{hɵ}\right)\times \;\left(1-{\mathrm{RDenit}}_{ɵ}\right),$$

(3) $${\mathrm{FdNF}}_{ɵ}=\sum_{h=1}^{d}\sum_{k=1}^{q}Y_{kɵ}({\mathrm{FdNF}\_\mathrm{Prod}}_{\mathrm{hk}ɵ})+\sum_{h=1}^{d}\sum_{k=1}^{q}Y_{kɵ}({\mathrm{FdNF}\_\mathrm{Cons}}_{\mathrm{hk}ɵ}),$$

where FdNF_Prod*_hk_* is the N footprint for the production of food item *h* for dietary group *k*, Prot_Sup*_hk_* is the per capita protein supply of food item *h* for dietary group *k* [27,33], Prot_Ncont*_h_* is the N content of protein supplied by food item *h* [34], FdWst*_h_* is the consumer-level food waste ratio of food item *h* [13,35], VNF_Dom*_h_* is the annual VNF of domestically produced food item *h*, FdNF_Cons*_hk_* is the N footprint for the consumption of food item *h* for dietary group *k*, RDenit is the ratio of denitrification (set to 0% because no report on wastewater treatment with N removal technology in India is available yet), FdNF*_ɵ_* is the total food N footprint, d (=35) is the total number of food items, q (=2) is the number of dietary groups, and *Y_k_* is the population ratio of dietary group *k*.

In the *P*-Calculator method, production and consumption parts are calculated from the supplied food (Fd_Sup), multiplied by the P content (Fd_Pcont), the proportion of food not wasted at the consumer level (1 − FdWst), and the corresponding factor for P loss during production (VPF_Dom). Within the same system boundaries, the food P footprint (FdPF) of state/union territory *ɵ* is estimated as the sum of the production part (FdPF_Prod) (Equation (4)) and the consumption part (FdPF_Cons) (Equation (5)), weighted by the vegetarian and non-vegetarian groups (Equation (6)):

(4) $${\mathrm{FdPF}\_\mathrm{Prod}}_{\mathrm{hk}ɵ}\;=\;{\mathrm{Fd}\_\mathrm{Sup}}_{\mathrm{hk}ɵ}\;\times \;{\mathrm{Fd}\_\mathrm{Pcont}}_{h}\;\times \;\left(1-{\mathrm{FdWst}}_{hɵ}\right)\;\times \;{\mathrm{VPF}\_\mathrm{Dom}}_{hɵ},$$

(5) $${\mathrm{FdPF}\_\mathrm{Cons}}_{\mathrm{hk}ɵ}={\mathrm{Fd}\_\mathrm{Sup}}_{\mathrm{hk}ɵ}\;\times \;{\mathrm{Fd}\_\mathrm{Pcont}}_{h}\;\times \;\left(1-{\mathrm{FdWst}}_{hɵ}\right),$$

(6) $${\mathrm{FdPF}}_{ɵ}=\sum_{h=1}^{d}\sum_{k=1}^{q}Y_{kɵ}({\mathrm{FdPF}\_\mathrm{Prod}}_{\mathrm{hk}ɵ})+\sum_{h=1}^{d}\sum_{k=1}^{q}Y_{kɵ}({\mathrm{FdPF}\_\mathrm{Cons}}_{\mathrm{hk}ɵ}),$$

where FdPF_Prod*_hk_* is the P footprint for the production of food item *h* for dietary group *k*, Fd_Sup*_hk_* is the per capita supply of food item *h* for dietary group *k* [27,33], Fd_Pcont*_h_* is the P content of food item *h* [34], VPF_Dom*_h_* is the annual VPF of domestically produced food item *h*, FdPF_Cons*_hk_* is the P footprint for the consumption of food item *h* for dietary group *k*, FdPF*_ɵ_* is the total food P footprint, d (=35) is the total number of food items, q (=5) is the number of dietary groups, and *Y_k_* is the population ratio of dietary group *k*.

### 2.3. Virtual Nitrogen and Phosphorus Factors

Factors for calculating N*r* and P losses in all the upstream production steps (N*r*Loss or PLoss) based on the consumed portion (NCons or PCons), VNFs and VPFs, are important parts of the footprint calculation. VNFs and VPFs are the sum of N*r* or P lost to the environment across six production steps [36]. Losses at each step are calculated from the ratio of the portion available in the next step—i.e., the N or P use efficiency (NUE or PUE)—along with the recycling ratio, and for N*r*, the ratio of removal as N_2_ gas. The VNF and VPF of each food item produced domestically in state/union territory *ɵ* in India were computed as follows (Equations (7) and (8)):

(7) $${\mathrm{VNF}\_\mathrm{Dom}}_{hɵ}\;=\;\frac{{Nr\mathrm{Loss}}_{hɵ}}{{\mathrm{NCons}}_{hɵ}},$$

(8) $${\mathrm{VPF}\_\mathrm{Dom}}_{hɵ}=\frac{{\mathrm{PLoss}}_{hɵ}}{{\mathrm{PCons}}_{hɵ},}$$

where VNF_Dom*_hɵ_* is the annual domestic virtual N factor of food item *h*, N*r*Loss*_h_* is the amount of N*r* lost to the environment during the production of food item *h* (from N input to final consumers, the loss includes the loss at the consumer level), NCons*_h_* is the amount of N consumed from food item *h* (N in the food eaten), VPF_Dom*_hɵ_* is the annual domestic virtual P factor of food item *h*, PLoss*_h_* is the amount of P lost to the environment during the production of food item *h* (from P input to final consumers, the loss includes the loss at consumer level), and PCons*_h_* is the amount of P consumed from food item *h* (P in the food eaten).

### 2.4. Nitrogen and Phosphorus Use Efficiencies

Among the six production steps, the cultivation step (crop uptake/release of the nutrient) is a major leakage step. Cultivation NUE and PUE of crop *r* produced in state/union territory *ɵ* in India were computed based on the N or P content in the harvested crop ($N_{\mathrm{cont}}$ or $P_{\mathrm{cont}}$) and inputs from chemical fertilizers ($N_{\mathrm{fert}}$ or $P_{\mathrm{fert}}$), manure ($N_{\mathrm{man}}$ or $P_{\mathrm{man}}$), atmospheric deposition ($N_{\mathrm{adep}}$ or $P_{\mathrm{adep}}$), seeds ($N_{\mathrm{seed}}$ or $P_{\mathrm{seed}}$), and for N, biologically fixed N ($N_{\mathrm{bfix}}$) as follows (Equations (9) and (10)):

(9) $${\mathrm{NUE}}_{rɵ}\;=\;\sum_{r=1}^{h}\frac{N_{{\mathrm{cont}}_{rɵ}}}{N_{{\mathrm{fert}}_{rɵ}}\;+\;N_{{\mathrm{man}}_{rɵ}}\;+\;N_{{\mathrm{adep}}_{rɵ}}\;+\;N_{{\mathrm{bfix}}_{rɵ}}\;+\;N_{{\mathrm{seed}}_{rɵ}}},$$

(10) $${\mathrm{PUE}}_{rɵ}=\sum_{r=1}^{h}\frac{P_{{\mathrm{cont}}_{rɵ}}}{P_{{\mathrm{fert}}_{rɵ}}+P_{{\mathrm{man}}_{rɵ}}+P_{{\mathrm{adep}}_{rɵ}}+P_{{\mathrm{seed}}_{rɵ}}},$$

where h (=15) is the total number of crops produced, $N_{\mathrm{cont}}$ is the N content in the harvested crop [37], $N_{\mathrm{fert}}$ is the N fertilizer applied [2], $N_{\mathrm{man}}$ is the N content in the livestock manure applied [1], $N_{\mathrm{adep}}$ is the atmospheric N deposition [38], $N_{\mathrm{bfix}}$ is the biological N fixation [39], $N_{\mathrm{seed}}$ is the N content in crop seed [13], $P_{\mathrm{cont}}$ is the P content in the harvested crop [34], $P_{\mathrm{fert}}$ is the P fertilizer applied [2], $P_{\mathrm{man}}$ is the P content in livestock manure applied [1], $P_{\mathrm{adep}}$ is the atmospheric P deposition [38], and $P_{\mathrm{seed}}$ is the P content in crop seed [40].

### 2.5. Auxiliary Sources of Data

Additional data sources used in the calculation include, fertilizer use by crop category [41], N efficiency in livestock production and slaughter, product collection efficiency, food and feed processing efficiency [13], feed composition and slaughter efficiency [42,43], food protein content [37], N to protein conversion factors [44], calorie to protein conversion factors [45], P efficiency in livestock production [46,47], P content in animal body parts [48], P efficiency in aquaculture production [49], livestock manure statistics [2], feed use [13], agricultural residue use [12], fisheries and aquaculture production [50], efficiency and recycling ratios of fish and seafood [36], food waste ratios [13,35], and protein intake [51].

### 2.6. Limitations of the Methodology

This study acknowledges several limitations that may impact the applicability and relevance of its findings. First, the data age: The footprint calculation used the 2011–2012 national statistics of India, corresponding to the latest census period at the time of analysis. While this dataset provides comprehensive and detailed official information, these data do not capture changes in economic growth, shifts in dietary patterns, and evolving agricultural practices that have likely occurred in India since then. Second, simplified dietary classification: Only two groups—vegetarians and non-vegetarians—were considered. This binary classification, while necessary for simplicity of modeling and tractability of the assessment, overlooks India’s rich dietary spectrum, including flexitarian habits, semi-vegetarianism, and intra-religious distinctions influenced by caste, region, or personal belief. These oversights may affect the accuracy of capturing individual food choices within India’s diverse cultural and religious context. Future studies could improve upon this by incorporating finer dietary typologies, such as frequency-based or caste-informed dietary groupings, using high-resolution survey data. Third, the scope limitations: Energy-related N and P footprints, such as emissions from food transportation and processing, were excluded. Including these aspects would provide a more holistic understanding of the environmental impacts associated with food systems and strengthen the relevance of policy recommendations.

## 3. Results

### 3.1. Regional Variations in Resource Use

Focusing on resource use, our analysis for 2011–2012 reveals significant regional variations in N and P consumption through food (Figure S2). The total N and P in the food consumed across India was 4115 Gg-N year^−1^ and 831 Gg-P year^−1^, respectively. Notably, Uttar Pradesh, the largest state, had the highest consumption (19.83 Gg-N year^−1^ and 3.97 Gg-P year^−1^), while Lakshadweep, the smallest union territory, had the lowest (0.01 Gg-N year^−1^ and 0.004 Gg-P year^−1^). Even on a per capita basis, Uttar Pradesh ranks in the top half of all the 28 states and territories, while Lakshadweep ranks in the bottom half.

There was a wide range of variation in the VNF and VPF, i.e., the loss of N and P relative to the content in the food consumed, among the states and union territories (Table 1 and Table 2). For instance, in agriculturally intensive Punjab, the VNF for cereals was only 1.80, reflecting highly efficient cereal production, whereas in a less efficient agrarian state like Odisha it was 14.9. The “other plant products” category shows the highest VNF values (often exceeding 40 in certain states and union territories such as Arunachal Pradesh, D. & N. Haveli, and Lakshadweep), indicating extremely high N losses for those foods, while categories like starchy roots and fruits generally had much lower VNFs (often below 1). Table 2 shows similar contrasts for phosphorus. Punjab’s cereal VPF was 1.80, compared to 19.19 in Odisha, and an extreme 42.38 for meat in Rajasthan—by far the highest P loss per unit intake—whereas many staple foods in certain states and union territories had VPFs approaching zero.

**Figure 3:**
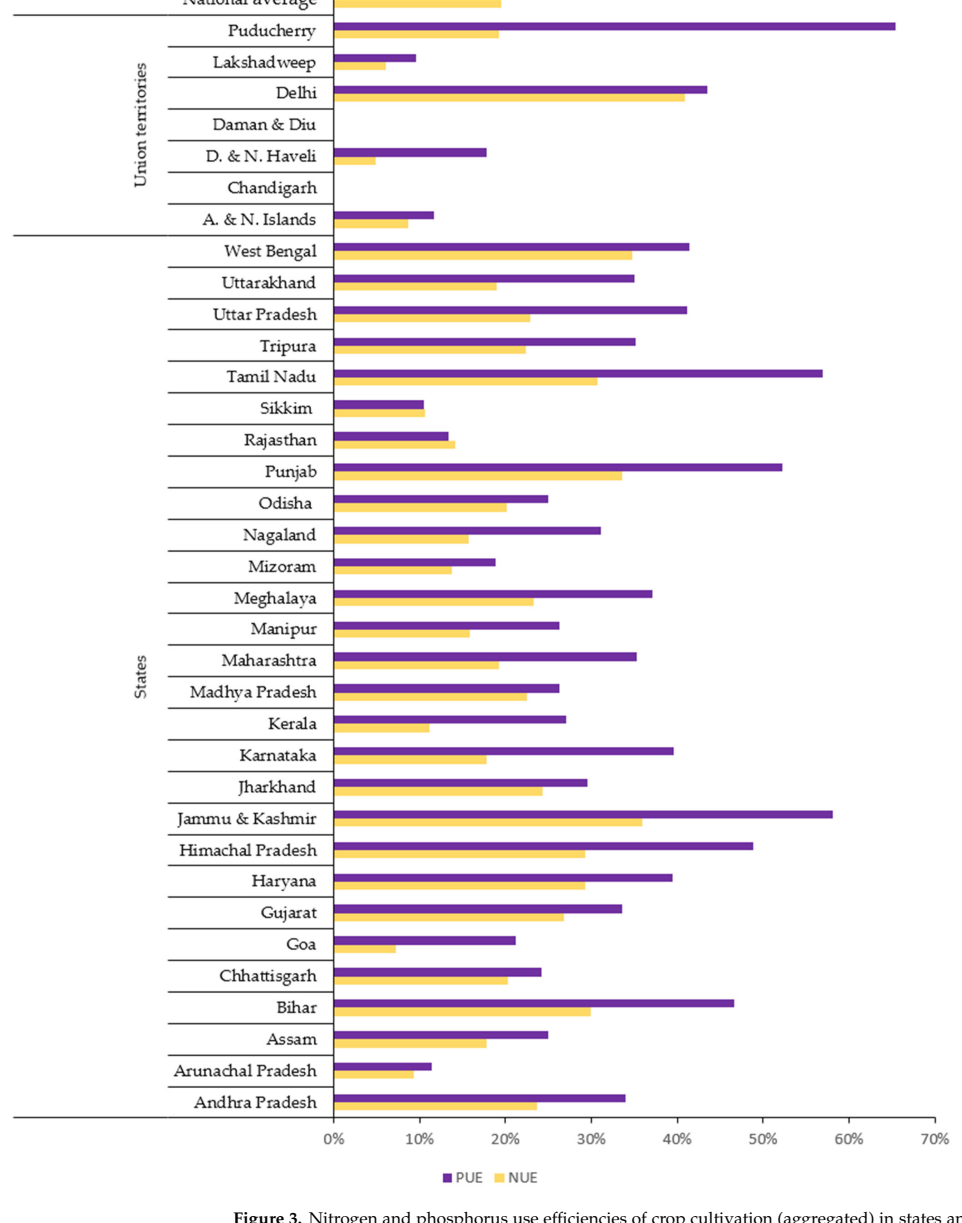
Nitrogen and phosphorus use efficiencies of crop cultivation (aggregated) in states and union territories of India during 2011–2012.

Decomposing the analysis, high VNFs and VPFs are largely attributed to low NUEs and PUEs in food production, especially in crop cultivation. This is because low NUEs and PUEs indicate that more N and P inputs are required to produce a unit of food, often leading to higher N and P footprints. However, with certain exceptions, such as systems with high recycling ratios, efficient reuse of N and P can reduce their losses and lower VNFs and VPFs, even with lower overall NUEs and PUEs [24]. During 2011–2012, the national average NUE of crop cultivation was 19% and the national average PUE of crop cultivation was 31%. Focusing on the state level NUEs and PUEs, only a few regions achieved crop cultivation NUE above 30% (and crop cultivation PUE above 50%), whereas many states and union territories remained at or below the national averages of 19% and 31%, respectively (Figure 3). Delhi and Puducherry had the highest NUE (41%) and PUE (65%) of crop cultivation, respectively, followed by Jammu & Kashmir, West Bengal, and Punjab for both N and P. In contrast, both the NUE and PUE of crop cultivation in Chandigarh and Daman & Diu were close to 0%. These variations reflect the diverse dietary habits and agricultural practices in different regions, highlighting the need for region-specific approaches to nutrient management.

### 3.2. Contributions of Food Groups to Resource Use

**Figure 4:**
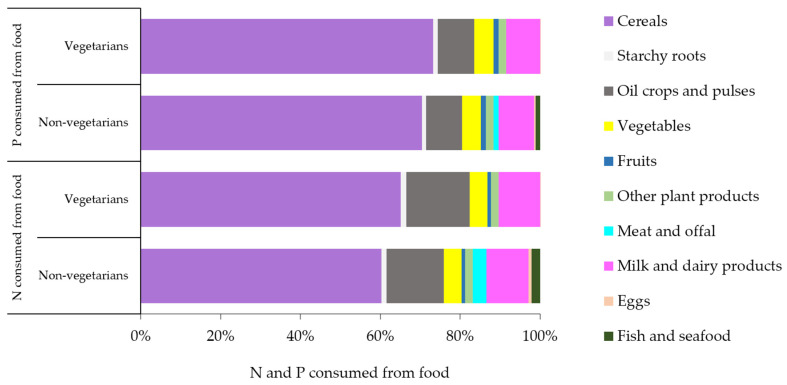
Nitrogen and phosphorus consumed from different foods by dietary groups in India during 2011–2012.

With a focus on the contributions of food groups, cereals, a staple food of the Indian diet, emerged as the major contributors to N and P consumption (Figure 4). This trend was particularly pronounced among vegetarians, who obtain N and P mainly from plant sources, especially cereals. The highest VNF and VPF values were recorded for “other plant products”, followed by cereals and “meat and offal”, while the lowest values were recorded for starchy roots, vegetables, and fruits. Legumes and oil crops also showed moderate VNF and VPF values, reflecting their role as significant protein and nutrient sources in vegetarian diets (Table 1 and Table 2). These values underline the environmental implications of their cultivation, particularly in regions with low NUE and PUE. On the other hand, animal-derived food products, while contributing to elevated footprints, were found to be regionally variable, influenced by dietary preferences and agricultural practices.

### 3.3. Diet-Based Differences in Food Nitrogen and Phosphorus Footprints

#### 3.3.1. Total Food Nitrogen and Phosphorus Footprints at Sub-National Level

**Figure 5:**
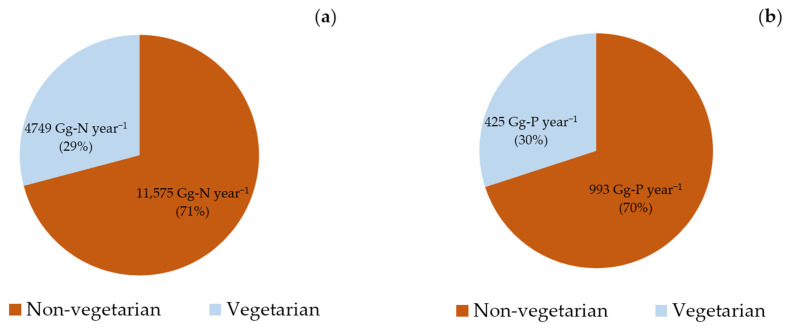
Total food (**a**) nitrogen and (**b**) phosphorus footprints by dietary groups in India during 2011–2012.

This study revealed that India’s total food N footprint in 2011–2012 was 16,324 Gg-N year^−1^ and the P footprint was 1418 Gg-P year^−1^. The total food N footprint at the state and union territory level varied from 2425 Gg-N year^−1^ (Uttar Pradesh) to 1 Gg-N year^−1^ (Lakshadweep), while the P footprint ranged from 194 Gg-P year^−1^ for Uttar Pradesh to ≥1 Gg-P year^−1^ for most union territories, except Delhi (18 Gg-P year^−1^; Figure S3a,b). Since the majority of the Indian population identifies as non-vegetarians, the total food N and P footprints of this dietary group (11,575 Gg-N year^−1^ and 993 Gg-P year^−1^, respectively) were higher than those of vegetarians (4749 Gg-N year^−1^ and 425 Gg-P year^−1^, respectively) (Figure 5a,b).

#### 3.3.2. Personal Food Nitrogen and Phosphorus Footprints

In 2011–2012, the average personal food N and P footprints in India were 13.11 kg-N capita^−1^ year^−1^ and 1.16 kg-P capita^−1^ year^−1^, respectively (Table 3). The personal food N and P footprints of non-vegetarians were significantly higher than those of vegetarians in all states and territories examined. In most areas, the personal food N and P footprints were higher than the national averages (Figure S4a,b).

Highlighting the regional diversity, the highest personal food N footprint was 18.88 kg-N capita^−1^ year^−1^ for Sikkim. This included a vegetarian footprint of 18.16 kg-N capita^−1^ year^−1^ and a non-vegetarian footprint of 18.98 kg-N capita^−1^ year^−1^. In contrast, the lowest personal food N footprint was 6.86 kg-N capita^−1^ year^−1^ for Chandigarh. This included a vegetarian footprint of 6.32 kg-N capita^−1^ year^−1^ and a non-vegetarian footprint of 7.96 kg-N capita^−1^ year^−1^. Similarly, for P, the highest personal food P footprint was 1.92 kg-P capita^−1^ year^−1^ in Odisha, including a vegetarian footprint of 1.88 kg-P capita^−1^ year^−1^ and a non-vegetarian footprint of 1.92 kg-P capita^−1^ year^−1^. On the other hand, the lowest personal food P footprint was 0.53 kg-P capita^−1^ year^−1^ in Daman and Diu, where the footprints of both vegetarians and non-vegetarians were the same, at 0.46 kg-P capita^−1^ year^−1^.

**Figure 6:**
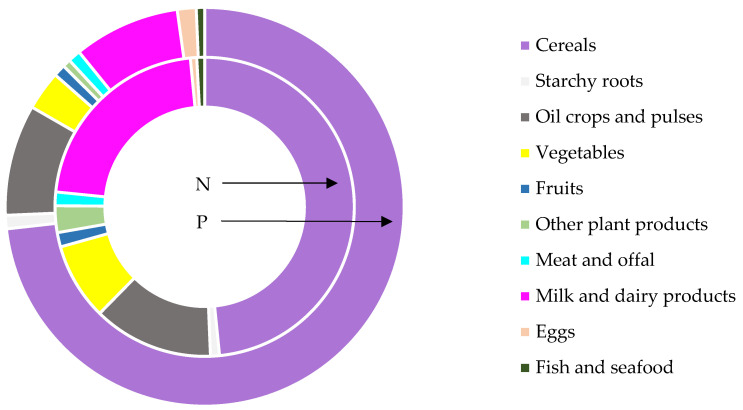
Overall contribution of food items to the nitrogen and phosphorus footprints of India during 2011–2012.

The contributions of different food groups to the overall personal food N and P footprints reveal significant patterns of resource use in India. Cereals made the largest contribution to the overall personal food N footprint of India, followed by “milk and dairy products”, whereas “fish and seafood” and eggs made the smallest contributions. After cereals, “oil crops and pulses” contributed the most to the overall Indian food P footprint, while “other plant products” contributed the least (Figure 6). The findings suggest that opting for a plant-based diet instead of an animal-based one can reduce N and P footprints both on an individual and regional level. Targeted strategies like culture- and religion-sensitive dietary interventions and improved nutrient management practices are particularly important in countries like India, where regional diversity significantly affects resource use patterns. This insight can contribute to the development of strategies aimed at not only minimizing the N and P footprints in India but also on a global scale, as elaborated in the following section.

In summary, the results demonstrate distinct regional disparities in food N and P footprints across India’s states and union territories (Table 4). For example, Sikkim’s food N footprint is nearly three times higher than that of Chandigarh, and Odisha’s food P footprint is over three times that of Daman & Diu. These contrasts reflect both differences in crop cultivation N and P use efficiencies, and dietary composition. Generally, regions with more efficient, intensive crop production and predominantly vegetarian diets (e.g., Punjab or Rajasthan) tend to exhibit lower VNF and VPF values, while those with less efficient farming practices or greater consumption of animal-based foods (e.g., parts of the Northeast and coastal states like Kerala) show markedly higher values. Table 3 illustrates this spectrum by comparing a set of representative states. Punjab, an agriculturally intensive and largely vegetarian state, achieves relatively high crop cultivation N and P use efficiencies, and correspondingly low VNFs and VPFs; whereas Kerala shows much higher N and P losses per unit of food consumed with a more fish- and other plant product-oriented diet, and lower crop cultivation N and P use efficiencies. Delhi, a heavily urbanized territory, benefits from importing food produced elsewhere, yielding moderate footprints despite high total consumption, while Uttar Pradesh, India’s most populous state, falls intermediate in efficiency and footprints.

## 4. Discussion

### 4.1. Plant-Based Diets and Religious Dietary Behaviors

Food N and P footprints in India are predominantly driven by dietary habits, which are largely the result of religious dietary practices [19]. These practices often entail the preferential use of specific foods under specific conditions, often giving symbolic value to certain foods. Conversely, certain foods are prohibited despite being freely available. Religious edicts also often restrict entire categories of foods from being consumed [52].

Vegetarianism explains a range of Indian dietary practices that involve the partial or total exclusion of animal-based foods [53]. Fruitarianism is known as the sternest form of vegetarianism, where followers consume only fruits [54]. Fruitarians, such as Jains, consider fruits as a harmless gift of nature. Strict Jains also avoid consuming dairy products [55]. Most vegan Buddhists and Sikhs avoid all animal products of animal origin, including honey, eggs, milk, and dairy products [56]. Many Hindus are lacto-vegetarians abstaining from animal meat but allowing eggs, milk, and dairy products as sources of protein [57]. While meat consumption in India, except in Rajasthan and Punjab, has increased over the past decade, high-caste, politically dominant Hindus have turned to vegetarianism. At least 23 states have now banned beef consumption, 15 states have prohibited eggs in school lunch, and some airlines have adopted largely vegetarian menus [58]. However, recent national survey analyses indicate that the share of Indian adults consuming meat has risen from about 74% in 2006 to nearly 80% by 2021, reflecting a gradual shift toward non-vegetarian food even as cultural influences remain strong [59].

These dietary choices and practices have significant implications for food N and P footprints in different Indian regions. The effects of increasing urbanization and rising income levels, which have led to a growing preference for animal-based diets [60,61,62], on the food N and P footprints should be considered in future studies. The binary classification of vegetarian and non-vegetarian diets, while emphasizing the environmental impacts of these groups, oversimplifies India’s complex dietary landscape. India’s cultural and religious diversity strongly influences dietary habits across states and union territories. Within these broad categories, substantial variations exist, such as differences in the types of meat consumed or the diversity of vegetarian diets shaped by regional and religious practices. Future studies should consider these nuances to provide a more accurate and comprehensive understanding of dietary impacts on the environment.

The total food N and P footprints of vegetarianism-dominated regions (e.g., Gujarat, Haryana, and Punjab) were much lower than those of non-vegetarianism-dominated regions (Figure S3a,b). Other states and union territories with low N and P footprints are areas with remarkably low agricultural productivity due to unfavorable agronomic conditions, hostile weather, inadequate availability of agricultural inputs, and uneven access to modern technology [63]. As noted by Dhar et al. (2021), the lower sub-national food N and P footprints of these areas are due to lower dependence on animal-based products and higher preference for plant-based products by the communities in these areas [19].

As demonstrated above, reducing food N and P footprints at the consumer level could be achieved by promoting plant-based diets, as plant-based foods typically have lower VNFs and VPFs compared to animal-based foods. The Indian government’s ‘Eat Right India’ campaign, and faith-based initiatives, such as annual national and international vegan festivals, already encourage plant-based diets to address climate change and promote sustainable living [64,65]. Global assessments continue to reinforce the benefits of plant-based diets for nutrient sustainability. A recent report by Leip et al. (2023) concluded that halving meat and dairy intake—paired with better farm management—could reduce nitrogen losses by roughly 50%, highlighting diet change as a key strategy for mitigating N pollution [66]. These findings support the notion that preserving and promoting plant-based dietary cultures in India could curb future growth in nutrient footprints, aligning environmental objectives with longstanding cultural practices.

This study also underscores the importance of integrating cultural and religious contexts into strategies for reducing food N and P footprints allowing for region-specific, culturally sensitive interventions that respect and harmonize with local traditions and nutritional needs. While a complete shift may be impractical due to cultural practices, nutritional needs, and existing dietary habits, promoting a healthy, low-footprint diet predominantly based on plant-based foods offers a feasible solution for both vegetarian and non-vegetarian. For instance, religion-sensitive adaptations of the Eat-Lancet planetary health diet, combined with improved crop cultivation NUEs and PUEs, have shown significant reductions in personal food N and P footprints [24,57]. These adjustments–such as reducing cereals, starchy roots, and dairy while increasing fruits, vegetables, oil crops, pulses, and other plant products–align with nutritional recommendations and minimizing environmental impacts. Improving crop cultivation NUEs and PUEs further benefits crop growers by reducing N*r* and P losses, thereby supporting both environmental sustainability and agricultural efficiency. Together, these strategies provide a balanced approach that supports diversity, environmental sustainability, and cultural sensitivity.

### 4.2. Status of Nitrogen and Phosphorus Use Efficiencies

The high food N and P footprints of several Indian states and union territories can be explained by the low soil N and P contents, overfertilization, and inefficient use of other energy inputs [57,67]. Among these factors, overfertilization is significant, resulting in the low crop cultivation NUEs and PUEs of states/union territories in India (Figure 3). Even in the vegetarian-dominated states/union territories with the lower total food N and P footprints described in Section 4.1, the personal footprints are still higher than in some areas; this is attributed to higher N*r* and P losses at the production level due to low NUEs and PUEs. From the 1960s onwards, the crop cultivation NUEs and PUEs have declined sharply in India and other South Asian countries [24]. In recent decades, advancements in agricultural practices, such as the adoption of precision farming technologies and shifts in fertilizer usage, may have altered NUE and PUE over the past decade [25,68]. Using forthcoming national statistics of India based on the 2024–2025 national census, these aspects can be addressed in future studies.

Globally, the crop production NUE in 2011–2012 averaged 50%, with developed regions such as North America and Europe achieving 59% and 49%, respectively. Asia’s average crop production NUE stood at 41%, while Africa had the highest crop production NUE at 73%. In contrast, India’s national crop production NUE was significantly lower at 19%. Similarly, global crop production PUE averaged 61%, with North America and Europe reaching 80% and 69%, respectively. Asia’s crop production PUE was 51%, and Africa’s exceeded 100% (135%) [69] due to the mining of phosphorus from soils in nutrient-depleted regions [70]. India’s national crop production PUE during the same period was substantially lower, at 31%. The major factors driving India’s low NUE and PUE at the sub-national level, leading to a low national average, include (a) the low uptake potential of nutrients by modern cultivars, (b) the need for large-scale fertilizer application, and (c) imbalanced nutrient application. Many high-yielding cultivars in India, while bred for productivity, exhibit low nutrient uptake potential, resulting in inefficient use of applied fertilizers [71]. Additionally, the widespread reliance on large-scale fertilizer application often exceeds crop requirements, leading to nutrient runoff and environmental degradation [72]. Only about 30–45% of applied N fertilizer and 15–25% of applied P fertilizer is actually taken up by crops in India, with the remainder lost to the environment [73]. The imbalance in nutrient application, such as excessive N and insufficient P, further reduces the efficiency of nutrient uptake, depleting soil health and diminishing crop productivity [74]. These differences underline the inefficiencies in nutrient management practices across Indian agriculture and highlight the need for targeted interventions to close this gap.

VNFs and VPFs are aggregated factors based on the N and P losses of each production step calculated from the NUEs and PUEs, respectively, the recycling ratio, and, for N*r*, the removal ratio (Section 2.3). Thus, the unbalanced and inefficient use of fertilizers not only reduces the NUEs and PUEs of crop cultivation, but also increases the total N and P losses per the final content, VNFs and VPFs, of animal-based foods. The highest values for both domestic VNF and VPF at the national level were found in “other plant products” (19.12 and 11.70, respectively) while the lowest were found in starchy roots (0.73 and 0.76, respectively) (Table 1 and Table 2). This indicates that for each unit of N and P intake from food, 0.73–19.12 units N*r* and 0.76–11.70 units P would be released into the environment.

High or low NUEs and PUEs of food production in some regions can indicate a state of food surplus or deficit at the sub-national level. Food waste has strong positive correlation with food surplus, which could lead to elevated N and P footprints [75,76]. It was observed that states with comparatively high food N and P footprints (i.e., Andhra Pradesh, Gujarat, Haryana, Karnataka, Madhya Pradesh, Maharashtra, Odisha, Punjab, Rajasthan, Tamil Nadu, Uttar Pradesh, and West Bengal) had food surpluses in 2011–2012. Food surpluses typically included meat and offal, milk and dairy products, cereals, and eggs (Figure S5a,b). Several states, including Bihar, Jharkhand, Kerala, Odisha, Rajasthan, Tamil Nadu, and Uttar Pradesh, were deficient in vegetables, oil crops and pulses; however, due to unfavorable geographical conditions for agricultural production, some states (e.g., Manipur, Meghalaya, Mizoram, and Nagaland) and all union territories were either food deficient or marginally food surplus. The large size and agro-climatic diversity of India entails that different crops are grown in different regions of the country. This leads to significant movement of food items among states and union territories. Haryana, Madhya Pradesh, and Punjab are the states with surplus wheat production. Andhra Pradesh, Chhattisgarh, Haryana, Odisha, and Punjab feature a surplus of rice. Consequently, the surpluses of wheat and rice in these states are transferred to deficit states to meet consumer demand under the National Food Security Act and other related schemes. More than 60% of the procured stocks are moved from the surplus regions to the deficit regions [77].

### 4.3. International Footprint Comparison

The total food N and P footprints in India estimated here are comparatively higher than those reported by Oita et al. (2020) [3] and Dhar et al. (2021) [19]. In this study, data on the sources of food production and consumption in each Indian state and union territory were obtained from the website of the Ministry of Program Implementation and Statistics, India, and the Ministry of Agriculture and Farmers Welfare, India. Those for Oita et al. (2020) [3] and Dhar et al. (2021) [19] were obtained from the FAO (2019) [2], to account for these differences. A similar study was conducted by McCourt and MacDonald (2021) [22] in Canada, wherein the authors quantified the largest N footprint from the province of Saskatchewan (50.30 kg-N capita^−1^ year^−1^) and the smallest from the province of Ontario (22.00 kg-N capita^−1^ year^−1^). Compared to the developing countries in South Asia, with the exception of Pakistan, India’s food N footprint is considerably higher than that of other countries in South Asia, while its food P footprint is similar to that of other countries [24,57]. However, when compared with the developed countries in Asia, the estimated results of this study are significantly lower than those for two East Asian countries, China, and Japan. Recent food N and P footprints for China have been estimated at 19–22 kg-N capita^−1^ year^−1^ [3,13,78] and 3–5 kg-P capita^−1^ year^−1^ [3,10], while those for Japan have been estimated at 15–28 kg-N capita^−1^ year^−1^ [3,12,39,79,80] and 3–6 kg-P capita^−1^ year^−1^ [3,10].

Further global comparisons help contextualize India’s footprint levels. Because specific food N and P footprints are not available for all countries, the total N and P footprints are used for comparison. The food N and P footprints of India are relatively close to those of Indonesia, a country in the Southeast Asia (13.7 kg-N capita^−1^ year^−1^ and 2.1 kg-P capita^−1^ year^−1^, respectively) [81]. Urban populations in rapidly developing regions tend to have greater nutrient footprints than their rural counterparts. Xia et al. (2020) [82] found that residents of metropolitan Shanghai in China had food N footprints in the range of 15.3–18.8 kg-N capita^−1^ year^−1^. Comparing the value range to 12.6–17.4 kg-N capita^−1^ year^−1^ in nearby rural areas, it reflects increased animal protein intake with urbanization [82]. In certain regions characterized by intensive livestock-based diets, per capita footprints can reach even higher levels; for instance, the Qinghai-Tibetan Plateau in western China exhibits average food N footprints on the order of 27.7 kg-N capita^−1^ year^−1^ [83]. These comparisons illustrate that nutrient footprints rise markedly when diets shift toward greater animal protein. It also reinforce that the lower consumption of animal-derived foods, a diet followed by much of the Indian population, is responsible for the much lower personal food N and P footprints in India than those of countries in North America, Australia, Europe, and in parts of Asia and Africa [22,84,85,86,87].

As of 2023, India is the most populous country of the world. To meet the dietary requirements of its 1.4 billion population, 666.4 Tg of N, 189.1 Tg of P, and 244.8 Tg of Potassium (K) were used during the period 1970–2018. This accounts for 68.1% of N, 91.3% of P, and 28.8% of K in chemical fertilizer inputs [88]. India is the largest producer of milk and pulses and ranks second in the production of rice, wheat, sugarcane, cotton, groundnuts, fruits, and vegetables [89]. However, a substantial quantity of food goes to waste, accounting for 8–10% of global greenhouse gas emissions [90]. Thus, the study of India’s food N and P footprints is important as it has a meaningful impact on both regional and population variations. In the decade following the base year of this study (2011–2021), India has experienced major dietary shifts influenced by economic growth and urbanization. Recent FAO food balance sheets indicate a noticeable rise in the consumption of animal-sourced foods. The per capita protein supply increased from about 60.50 g day^−1^ in 2011 to about 70.50 g day^−1^ in 2021, with the share of protein derived from animal products rising from around 19% to 25% [91]. Animal-based foods now contribute approximately 36% of per capita dietary fat, compared with about 31% a decade earlier. National surveys also confirm higher intake of milk, eggs, fish, and meat across all regions and income groups, along with a gradual decline in cereal-based diets [92]. These developments suggest that the food N and P footprints found from this study underestimate current footprints, as more animal-based food intake generally amplifies N and P losses. This underscores the urgency to improve nutrient use efficiency and promote sustainable dietary practices as India’s food system continues to evolve.

### 4.4. Toward Sustainable Production and Consumption

Minimizing N and P losses at both production and consumption levels is crucial to reducing the food N and P footprints in India, and achieving sustainable and equitable socioenvironmental development [3]. As described above, one of the most urgent challenges at the level of state/territorial food production is to reduce N and P losses to the environment from crop cultivation by increasing NUEs and PUEs. The inefficiencies in nutrient use are often impaired by socio-economic factors such as limited access to modern agricultural technology, insufficient farmer education, inadequate infrastructure, and fragmented government policies [93,94]. Addressing these issues is essential for creating practical and impactful solutions. For instance, investing in regional infrastructure, such as better irrigation systems and storage facilities, increasing access to precision farming technologies, and providing targeted training programs for farmers could significantly improve NUE and PUE [95,96,97]. Furthermore, strengthening policy frameworks to promote sustainable fertilizer application and incentivize sustainable agricultural practices can help mitigate nutrient losses [98,99].

At the level of food consumption, raising awareness of the importance of a low-VNF/VPF diets with foods that reduce N and P losses per unit of N and P consumed would necessarily lower food N and P footprints. In addition to these, it is also important to introduce efficient food waste management as food waste amplifies food N and P footprints by increasing the amount of N*r* and P lost across the food supply chain [100]. It has been reported that the Indian population wastes as much food as the entire consumption of the United Kingdom [101], with most wasted food coming from hotels and restaurants, supermarkets, residential areas, airline cafeterias, and food processing plants [102]. Intermittent food systems and weak supply logistics entail that 40% of food produced in India each year is wasted, equivalent to 50 kg of waste per person per year [90,103]. Food waste at the consumer level accounts for 3.7% of the food N footprint and 3.9% of the food P footprint of domestically produced food in India [19]. Reducing food waste in India is critical, as it constitutes a significant component of virtual N and P loss, with digestate containing N ranging 1.1–9.6 kg-N t^−1^ [104], and resulting in a total virtual N loss of 22.8 Gg-N.

Much of the current food waste in India’s urban areas is sent for composting to produce biofertilizers, but a considerable amount is also dumped in landfills. In rural areas, farmers typically use wasted food as animal feed, but this practice has a history of transmitting diseases to both animals and humans. In 2017, the Indian government took the step of regulating food waste in hotels and restaurants, and during cultural occasions, but this did not address shortcomings in the retail sector [105]. It has thus become urgent to recycle food waste in a holistic manner, tailoring such processes at the sub-national level based on the type of waste generation and the attitude and awareness towards the environmental sustainability. Initiatives could include expanding infrastructure for waste segregation and recycling, promoting community-led composting programs, and incentivizing food rescue organizations to redistribute surplus food [106,107]. These domestic actions coincide with international commitments urging better nutrient stewardship. Notably, the Colombo Declaration under UNEP has set a target of halving nitrogen waste by 2030, emphasizing dietary change and improved waste management as key pillars [108]. Similarly, the G20 forum in 2023 included calls for its member countries (including India) to establish explicit food loss and waste reduction targets and adopt a “Target–Measure–Act” approach to curb waste [109]. Aligning India’s strategies with such global best practices can create synergistic benefits by reducing avoidable N and P losses while simultaneously improving food security and progress toward climate goals.

This study underscores the importance of considering cultural, religious, and regional differences when developing strategies to reduce food N and P footprints. These socioeconomic and cultural factors must be integrated into policy design to ensure practical and equitable solutions. Understanding these dynamics at the sub-national level, coupled with targeted policies, technological advancements, and community engagement, can significantly contribute to sustainable food systems in India. In addition, addressing energy-related aspects, such as emissions from food transportation and processing, would enhance the accuracy and relevance of the findings. Incorporating more recent data to reflect contemporary dietary trends and agricultural practices, as well as conducting finer-scale examinations of diverse dietary patterns and regional variations in resource use, could provide broader insights into India’s food system. These improvements would not only strengthen sustainable production and consumption strategies but also ensure policy recommendations are more closely aligned with current conditions and needs.

### 4.5. Policy Recommendations for Reducing Food Nitrogen and Phosphorus Footprints

Recent analyses of India’s administrative policies on nutrients highlight the need for more proactive approaches, particularly in light of the country’s significant N and P footprints [110]. These challenges are compounded by the country’s rapidly evolving food systems, diverse agricultural practices, and broad regional and cultural diversity. Effective nutrient management must account for the country’s heterogeneity by distinguishing between food-surplus and food-deficit states, addressing rural and urban disparities in waste generation and infrastructure, and considering religious as well as dietary factors that influence consumption patterns. Rather than applying uniform approaches, India needs tailored strategies that can improve N and P use efficiency, minimize environmental losses, and promote the transition toward sustainable food systems. This section offers detailed and actionable policy recommendations that align with India’s socio-cultural context and sub-national realities to foster more equitable and effective nutrient governance.

In regions with food surpluses, fertilizer subsidies often encourage excessive application, leading to nutrient runoff and environmental degradation. Policies should focus on optimizing fertilizer use through educating the farmers about the “4R Nutrient Stewardship” framework—applying fertilizers in the right source, at the right rate, at the right time, and in the right place [111]. Precision agriculture tools, such as soil nutrient mapping and global positioning system (GPS)-guided fertilizer application, should be made accessible to farmers through subsidies and targeted training programs [112]. Furthermore, fertilizer subsidy reforms should encourage the adoption of organic manure, biofertilizers, and enhanced-efficiency fertilizers to minimize N and P losses [113]. Conversely, in regions with food deficits, limited availability of fertilizers, high costs, and a lack of extension services hinder effective nutrient use [114]. Policies should prioritize capacity building through agricultural extension services [115], equipping farmers with knowledge on sustainable N and P management. Ensuring the availability of affordable fertilizers and organic inputs, such as compost and biofertilizers, can help improve soil fertility and crop productivity [116]. In addition, selecting strategic genetically improved crop varieties, following conservation agriculture principles (i.e., zero/minimum tillage, diversified crop rotation, and residue retention), intercropping cereals and legumes, and ensuring effective microbial inoculation are also recommended for enhancing crop cultivation NUEs and PUEs [116,117,118]. These measures should be complemented by investments in infrastructure, including irrigation systems and post-harvest storage facilities, to reduce N and P losses and enhance resilience [119,120]. Integration of crop and animal production as a means to re-use crop and animal waste would also increase NUEs and PUEs at the food production level [100].

Urban areas are well-known to generate large amounts of food waste [106], contributing to elevated N and P footprints. Policy interventions should prioritize developing infrastructure for waste segregation, composting, and biogas production. Establishing centralized composting facilities and promoting urban biogas plants can convert food waste into biofertilizers and renewable energy, thereby reducing environmental impacts. Food redistribution networks, facilitated through public–private partnerships, can redirect edible food waste to underserved populations [121,122,123]. In rural areas, community-led composting programs tailored to local waste types can offer a sustainable alternative, promoting N and P recycling while reducing dependence on chemical fertilizers. Encouraging the adoption of decentralized composting models can help rural communities to manage waste effectively, improve soil health, and foster sustainable agricultural practices [124,125].

In vegetarian-dominated regions, promoting diversified plant-based diets that incorporate underutilized crops, such as pulses and oil seeds [126], can enhance nutritional adequacy while reducing environmental impacts. In contrast, non-vegetarian-dominated regions should prioritize balanced dietary shifts that reduce red meat consumption while promoting low-impact protein sources, such as fish and pulses [57]. Policymakers should work with community leaders and local organizations to design culturally sensitive initiatives, and ensure that dietary interventions align with local cultural and religious practices to maximize acceptance and effectiveness.

State governments should develop region-specific nutrient management plans that integrate agro-climatic conditions, cropping systems, and socio-economic factors. Establishing regional N and P footprint benchmarks and conducting regular assessments can provide measurable progress indicators and inform adaptive management strategies. These policy recommendations provide a roadmap for reducing food N and P footprints at the sub-national level in India. By addressing region-specific challenges and opportunities, these strategies can contribute to the development of sustainable food systems that balance environmental goals with cultural and nutritional needs. This approach not only benefits India but also serves as a model for other countries seeking to align agricultural practices with sustainability objectives.

Overall, the discussion above highlights the multifaceted drivers of food N and P footprints in India, encompassing dietary preferences, agricultural inefficiencies, regional production-consumption disparities, and emerging urbanization trends. Plant-based diets, though historically aligned with cultural and religious norms, offer substantial environmental advantages that can be reinforced through targeted policy support. Concurrently, the low crop cultivation NUEs and PUEs in many Indian states and union territories demand urgent attention through technological, infrastructural, and educational interventions. International comparisons underscore India’s relatively lower per capita footprints due to low animal-based food consumption, yet the country’s aggregate environmental burden remains significant due to its population size. Furthermore, the rising intake of animal products and ongoing urban transitions suggest that nutrient footprints may escalate in the coming years without corrective action. Hence, integrating socio-cultural insights, sub-national heterogeneity, and contemporary consumption trends is imperative for designing effective, equitable, and forward-looking sustainability strategies.

## 5. Conclusions

This study has marked an important step in quantifying state- and union territory-specific food N and P footprints in India, considering the diverse dietary patterns that include both vegetarian and non-vegetarian preferences. The findings support the study’s presumption that areas with predominance of vegetarian diets have lower footprints compared to areas with a higher consumption of animal-based foods. This highlights the significant environmental impact of dietary choices at both individual and collective levels. However, it is crucial to recognize that the elevated food N and P footprints in certain areas are not solely due to dietary habits. Inefficiencies in nutrient use efficiency (NUE and PUE) also plays a significant role. In addition, ineffective food waste management exacerbates the challenge of sustainable nutrient management. Addressing these issues requires a multi-faceted approach. Optimizing fertilization practices, improving crop cultivation NUE and PUE through technological advancement, and implementing effective food waste strategies are essential. These interventions not only reduce the food N and P footprints, but also contribute to the broader goal of sustainable development.

From a policy perspective, the study emphasizes the need for regionally adapted nutrient management strategies that reflect local dietary and agronomic realities. In vegetarian-dominated regions, promoting diversified and underutilized plant-based foods can reinforce sustainability gains, while in non-vegetarian-dominated areas, shifting toward lower-impact animal protein sources may reduce environmental burdens. Policymakers should prioritize educational outreach, precision agriculture adoption, fertilizer subsidy reforms, and decentralized waste recycling infrastructure. Tailoring interventions to cultural and regional contexts offers a pathway for achieving nutrient stewardship while honoring India’s socio-cultural complexity. From a global perspective, India’s significant role in the global agri-food system highlights how strategies to reduce its food N and P footprints can serve as a model for other countries facing similar challenges. This supports sustainable food system transformation both within India and in nations striving to balance environmental protection with growing dietary diversity.

This study is subject to several limitations. First, the analysis is based on data from 2011–2012, which may not fully capture recent trends in dietary transitions and agricultural intensification. Second, the dietary classification relied on a binary distinction between vegetarian and non-vegetarian groups, which does not reflect the complexity of Indian dietary behaviors. Third, the scope was limited to direct food-related N and P losses and did not account for energy-related emissions. Future research should incorporate more granular dietary classifications, update estimates using recent consumption and agricultural data, and expand system boundaries to include upstream and downstream nutrient flows for a more comprehensive assessment.

## Figures and Tables

**Table 1:** Domestic virtual nitrogen factors of states and union territories of India in 2011–2012.

States/Union Territories		Virtual Nitrogen Factors (kg-N Released per kg-N in Food Intake) by Aggregated Food Categories									
		Cereals	Starchy Roots	Oil Crops and Pulses	Vegetables	Fruits	Other Plant Products	Meat and Offal	Milk and Dairy Products	Eggs	Fish and Seafood
States	Andhra Pradesh	6.74	0.56	6.79	2.95	1.96	17.53	9.66	4.28	1.28	6.34
	Arunachal Pradesh	9.44	2.10	6.01	8.71	3.80	46.19	9.88	4.05	1.14	5.92
	Assam	10.73	1.53	8.66	2.43	1.80	14.22	9.50	4.35	1.76	8.70
	Bihar	5.69	0.43	3.12	2.30	1.76	13.31	9.44	4.47	1.24	5.66
	Chhattisgarh	8.41	0.79	6.36	2.42	2.51	13.77	9.36	4.23	1.28	7.91
	Goa	2.68	0.05	5.20	8.21	2.13	42.83	9.62	4.11	1.42	4.20
	Gujarat	9.84	0.28	2.15	3.55	0.91	20.22	9.62	4.81	1.21	7.28
	Haryana	7.62	0.33	3.05	2.54	4.32	17.84	9.65	4.67	1.28	8.36
	Himachal Pradesh	6.48	0.89	8.34	0.92	3.22	7.20	9.39	4.41	1.39	6.59
	Jammu & Kashmir	6.43	0.49	5.98	0.58	2.24	4.24	9.49	4.60	1.17	6.63
	Jharkhand	9.22	0.84	6.21	1.72	3.89	12.71	9.47	4.81	1.31	8.11
	Karnataka	8.54	1.53	8.46	4.14	1.65	24.97	9.45	4.47	1.31	8.57
	Kerala	3.60	1.04	10.96	6.20	5.88	34.64	9.53	4.67	1.31	5.62
	Madhya Pradesh	10.30	0.49	4.50	3.68	1.02	20.74	9.60	4.74	1.24	8.80
	Maharashtra	11.75	0.60	5.21	3.98	3.20	24.11	9.62	4.88	1.35	8.35
	Manipur	4.89	0.05	4.12	2.91	3.98	16.11	9.53	4.54	1.28	6.76
	Meghalaya	5.04	1.52	5.65	1.35	3.53	8.29	9.68	4.81	1.28	5.54
	Mizoram	8.49	1.14	3.53	3.35	3.76	18.05	9.61	4.60	1.24	8.49
	Nagaland	6.55	2.13	5.62	4.94	1.94	25.78	9.59	4.54	1.24	7.81
	Odisha	14.91	0.82	5.77	2.47	5.36	18.12	10.32	4.47	1.31	8.29
	Punjab	1.80	0.19	3.11	2.36	1.51	12.39	9.54	4.54	1.21	5.77
	Rajasthan	12.91	1.02	4.97	1.58	3.26	20.02	9.25	4.81	1.31	8.21
	Sikkim	11.97	4.25	4.86	4.43	4.29	23.96	10.14	4.67	1.24	8.47
	Tamil Nadu	2.46	0.44	2.86	3.47	0.72	17.45	9.64	4.54	1.14	6.15
	Tripura	2.23	0.55	9.56	1.38	4.17	9.29	9.30	4.54	1.31	5.62
	Uttar Pradesh	4.50	0.23	4.49	4.39	1.58	23.05	9.33	4.67	1.28	5.33
	Uttarakhand	5.53	0.52	4.80	4.18	9.20	21.98	9.28	4.74	1.24	6.90
	West Bengal	3.74	0.25	4.54	1.10	1.80	6.48	9.57	4.60	1.35	5.06
Union territories	A. & N. Islands	3.07	0.05	2.62	3.95	3.57	25.84	9.23	4.47	1.31	5.21
	Chandigarh	0.15	0.05	0.14	0.09	0.07	0.52	10.40	4.54	1.28	5.36
	D. & N. Haveli	3.25	0.05	4.17	8.85	0.07	43.96	8.93	4.41	1.21	6.94
	Daman & Diu	0.15	0.05	0.14	0.09	0.07	0.52	9.58	4.67	1.14	5.17
	Delhi	2.67	0.32	1.79	1.05	1.87	5.76	9.64	4.54	1.24	5.20
	Lakshadweep	0.15	0.05	2.79	8.58	9.20	43.71	9.64	4.60	1.24	6.99
	Puducherry	2.36	0.05	0.95	2.61	0.33	13.33	9.40	4.47	1.17	5.12
National level		6.12	0.73	4.79	3.36	2.87	19.12	9.57	4.55	1.28	6.73

**Table 2:** Domestic virtual phosphorus factors of states and union territories of India in 2011–2012.

States/Union Territories		Virtual Phosphorus Factors (kg-P Released per kg-P in Food Intake) by Aggregated Food Categories									
		Cereals	Starchy Roots	Oil Crops and Pulses	Vegetables	Fruits	Other Plant Products	Meat and Offal	Milk and Dairy Products	Eggs	Fish and Seafood
States	Andhra Pradesh	7.42	0.59	7.00	0.93	1.29	7.96	8.10	0.99	7.36	5.04
	Arunachal Pradesh	9.85	2.29	5.58	5.56	7.70	10.94	30.61	0.85	6.26	5.33
	Assam	14.18	1.74	11.82	1.20	1.10	14.78	9.82	0.97	6.71	6.91
	Bihar	5.08	0.41	4.04	0.63	0.90	7.96	5.59	0.96	6.56	6.08
	Chhattisgarh	8.41	0.88	8.22	1.53	1.83	14.60	10.36	0.93	6.12	4.28
	Goa	1.49	0.05	1.81	1.40	1.59	14.87	7.72	1.10	7.54	4.11
	Gujarat	11.25	0.35	4.04	1.20	0.57	7.96	9.90	0.90	5.72	4.09
	Haryana	8.85	0.42	4.72	0.64	3.50	14.70	10.84	1.02	6.41	6.20
	Himachal Pradesh	3.95	0.61	3.19	0.22	3.18	7.96	5.73	1.05	5.85	4.59
	Jammu & Kashmir	4.76	0.31	4.29	0.06	3.79	14.80	2.59	0.88	5.59	7.91
	Jharkhand	9.66	0.87	3.90	1.03	2.79	14.86	15.95	1.00	6.12	6.25
	Karnataka	8.90	1.45	4.34	0.58	0.88	7.96	6.14	0.93	5.34	6.42
	Kerala	2.21	1.56	3.12	1.02	5.20	7.96	5.77	1.02	6.56	4.93
	Madhya Pradesh	10.52	0.58	3.48	1.91	0.58	14.53	15.24	0.87	5.22	7.14
	Maharashtra	11.16	0.64	7.38	0.65	1.80	7.96	9.10	0.90	5.59	6.28
	Manipur	5.07	0.05	1.70	0.90	2.13	14.84	5.98	0.96	6.41	7.66
	Meghalaya	5.14	1.48	4.89	0.49	1.72	7.96	4.02	1.00	5.47	5.80
	Mizoram	8.51	1.15	1.38	2.27	2.12	7.96	11.88	0.91	6.56	5.64
	Nagaland	5.18	1.80	9.77	0.96	0.75	14.87	8.17	0.97	5.47	4.18
	Odisha	19.19	0.93	4.28	1.44	3.65	7.96	13.29	0.93	6.12	6.83
	Punjab	1.80	0.22	1.99	0.39	0.74	7.96	2.92	0.90	5.34	5.20
	Rajasthan	14.38	1.21	6.39	6.27	2.28	14.80	42.38	1.00	5.72	7.97
	Sikkim	11.55	4.07	0.14	3.47	2.31	14.53	17.32	0.93	5.59	7.15
	Tamil Nadu	2.78	0.48	1.51	0.36	0.29	7.96	1.79	0.97	5.34	4.05
	Tripura	1.12	0.64	5.56	0.57	2.51	14.58	4.13	1.02	6.56	4.33
	Uttar Pradesh	5.00	0.24	2.14	0.63	0.92	14.42	10.88	0.99	5.72	5.31
	Uttarakhand	6.19	0.55	2.31	0.66	6.17	14.76	4.48	1.03	5.34	5.28
	West Bengal	3.51	0.31	6.48	0.58	1.14	7.96	5.00	0.99	5.85	4.96
Union territories	A. & N. Islands	1.85	0.05	3.23	3.57	2.89	14.29	19.06	0.96	5.47	3.29
	Chandigarh	0.17	0.05	0.14	0.09	0.07	14.89	0.54	0.99	5.98	2.14
	D. & N. Haveli	1.68	0.05	3.00	1.31	0.07	14.72	6.63	1.05	6.56	3.67
	Daman & Diu	0.17	0.05	0.14	0.09	0.07	14.79	0.54	0.91	6.12	4.25
	Delhi	2.27	0.41	0.14	0.61	1.58	14.70	2.99	0.91	5.22	4.10
	Lakshadweep	0.17	0.05	−0.14	0.50	1.80	7.96	2.47	1.00	4.99	2.00
	Puducherry	1.19	0.05	−0.63	0.10	0.07	7.96	0.28	0.93	6.12	3.28
National level		6.12	6.13	0.76	3.75	1.25	2.00	11.70	9.09	0.96	5.97

**Table 3:** State and union territory-level food nitrogen and phosphorus footprints of India in 2011–2012.

States/Union Territories	Food Nitrogen Footprint (kg-N Capita^−1^ Year^−1^)			Food Phosphorus Footprint (kg-P Capita^−1^ Year^−1^)		
	Non-Vegetarian	Vegetarian	State/Union Territory Average	Non-Vegetarian	Vegetarian	State/Union Territory Average
Andhra Pradesh	14.28	13.61	14.27	1.20	1.11	1.20
Arunachal Pradesh	17.85	16.92	17.81	1.59	1.48	1.58
Assam	16.59	15.76	16.42	1.43	1.32	1.41
Bihar	11.87	11.19	11.82	1.10	1.07	1.10
Chhattisgarh	15.68	14.93	15.54	1.53	1.49	1.52
Goa	12.07	11.07	11.96	1.20	1.04	1.18
Gujarat	15.15	13.40	14.08	1.12	1.06	1.09
Haryana	12.49	10.37	11.01	1.05	0.96	0.98
Himachal Pradesh	14.43	13.22	13.79	1.33	1.25	1.29
Jammu & Kashmir	13.59	12.79	13.34	1.27	1.15	1.23
Jharkhand	13.84	13.23	13.82	1.23	1.18	1.22
Karnataka	15.60	14.71	15.41	1.09	1.02	1.08
Kerala	13.52	12.70	13.49	1.34	1.22	1.34
Madhya Pradesh	16.47	15.07	15.76	1.40	1.33	1.37
Maharashtra	17.71	16.53	17.24	1.50	1.42	1.47
Manipur	11.67	10.93	11.59	0.99	0.95	0.98
Meghalaya	11.67	11.06	11.64	0.96	0.90	0.96
Mizoram	13.50	12.79	13.46	1.68	1.61	1.67
Nagaland	15.14	14.39	15.13	1.07	1.02	1.07
Odisha	18.69	18.02	18.68	1.92	1.88	1.92
Punjab	10.97	9.07	9.70	0.85	0.79	0.81
Rajasthan	17.45	15.06	15.66	1.93	1.70	1.75
Sikkim	18.98	18.16	18.88	1.42	1.37	1.42
Tamil Nadu	11.15	10.47	11.13	0.81	0.75	0.81
Tripura	11.35	10.72	11.30	1.09	1.01	1.08
Uttar Pradesh	12.76	11.43	12.14	1.00	0.94	0.97
Uttarakhand	13.70	12.75	13.44	1.29	1.24	1.28
West Bengal	11.41	10.82	11.40	0.95	0.86	0.95
A. & N. Islands	11.05	10.29	10.98	1.20	1.06	1.19
Chandigarh	7.96	6.32	6.86	0.73	0.64	0.67
D. & N. Haveli	15.61	13.32	14.21	0.92	0.86	0.88
Daman & Diu	6.39	6.39	6.91	0.46	0.46	0.53
Delhi	10.50	9.54	10.12	1.12	1.06	1.09
Lakshadweep	10.33	0.00	10.33	0.72	0.00	0.72
Puducherry	9.65	9.01	9.64	0.88	0.79	0.88
National level	13.46	12.17	13.11	1.18	1.08	1.16

**Table 4:** Domestic virtual nitrogen and phosphorus factors, crop cultivation nitrogen and phosphorus use efficiencies, and food nitrogen and phosphorus footprints in selected Indian regions in 2011–2012.

States/Union Territories	Range of Virtual Nitrogen Factors (kg-N Released per kg-N in Food Intake)		Range of Virtual Phosphorus Factors (kg-P Released per kg-P in Food Intake)		Nitrogen Use Efficiency (%)	Phosphorus Use Efficiency (%)	Food Nitrogen Footprint (kg-N Capita^−1^ Year^−1^)	Food Phosphorus Footprint (kg-P Capita^−1^ Year^−1^)
	Plant-Derived	Animal-Derived	Plant-Derived	Animal-Derived				
Delhi	0.32–5.76	1.24–9.64	0.14–14.70	0.91–5.22	41	44	10.12	1.09
Kerala	1.04–34.64	1.31–9.53	1.02–7.96	1.02–6.56	11	27	13.49	1.34
Punjab	0.19–12.39	1.21–9.54	0.22–7.96	0.90–5.34	34	52	9.70	0.81
Uttar Pradesh	0.23–23.05	1.28–9.33	0.24–14.42	0.99–10.88	23	41	12.14	0.97
National level	0.73–19.12	1.28–9.57	0.76–6.13	0.96–11.70	19	31	13.11	1.16

## Data Availability

The original contributions presented in this study are included in the article/Supplementary Materials. Further inquiries can be directed to the corresponding authors. Data available on request due to restrictions on redistributing aggregated datasets derived from multiple data sources.

## Supplementary Materials

The following supporting information can be downloaded at: https://www.mdpi.com/article/10.3390/nu17233758/s1, Figure S1: State- and union territory-level (**a**) population by religion and (**b**) dietary choice by population in India in 2011–2012; Figure S2: Total nitrogen and phosphorus consumed from food in states and union territories of India during 2011–2012; Figure S3: Total food (**a**) nitrogen and (**b**) phosphorus footprints in states and union territories of India during 2011–2012; Figure S4: Differences between state- and union territory-level food (**a**) nitrogen and (**b**) phosphorus footprints relative to the national average during 2011–2012; Figure S5: Surplus/deficit in (**a**) aggregated food categories and (**b**) eggs in states and union territories of India during 2011–2012. Reference [127] is cited in the Supplementary materials.

## Abbreviations

The following abbreviations are used in this manuscript:

A. & N. Islands	Andaman and Nicobar Islands
D. & N. Haveli	Dadra and Nagar Haveli
et al.	et alia (and others)
Gg	Gigagram
GHG	Greenhouse gas
GPS	Global positioning system
i.e.,	id est (that is)
K	Potassium
kg	Kilogram
N	Nitrogen
N*r*	Reactive nitrogen
NUE	Nitrogen use efficiency
N_2_	Dinitrogen
N_2_O	Nitrous oxide
P	Phosphorus
PUE	Phosphorus use efficiency
t	Ton
Tg	Teragram
VNF	Virtual nitrogen factor
VPF	Virtual phosphorus factor
&	And
%	Percentage
