# Supplementary material for: Dietary Influences on Nitrogen and Phosphorus Footprints in Indian Food Systems: A State and Union Territory-Level Analysis
Source: Nutrients. 2025 Nov 29;17(23):3758. doi: 10.3390/nu17233758 (PMC12694435; doi:10.3390/nu17233758)
Supplement: Supplementary file 1 [file nutrients-17-03758-s001.zip › nutrients-3921655-supplementary.pdf]

Supplementary Material

Titlle:

Dietary Influences on Nitrogen and Phosphorus Footprints in Indian Food Systems: A State and Union Territory-level Analysis

Authors:
Aurup Ratan Dhar [1,4], Azusa Oita [2,1,*], Himadri Kaushik [3], Ananta Narayan Panda [3], Tapan Kumar Adhya [3,*] and Kazuyo Matsubae [4,1]

Affiliations:

1 Research Institute for Humanity and Nature, 457-4 Kamigamo Motoyama, Kita-ku, Kyoto 603-8047, Japan; aurup971@gmail.com (A.R.D.); oita.azusa855@naro.go.jp (A.O.); kazuyo.matsubae.a2@tohoku.ac.jp (K.M.)
2 Institute for Agro-Environmental Sciences, National Agriculture and Food Research Organization, 3-1-3, Kannondai, Tsukuba 305-8604, Japan; oita.azusa855@naro.go.jp (A.O.)
3 School of Biotechnology, Kalinga Institute of Industrial Technology (Deemed University), Bhubaneswar 751024, India; himadrikaushik18@gmail.com (H.K.); anpandabiotek@gmail.com (A.N.P.); adhyas@yahoo.com (T.K.A.)
4 Graduate School of Environmental Studies, Tohoku University, 468-1 Aoba, Aramaki, Aoba-ku, Sendai, Miyagi 980-0845, Japan; aurup971@gmail.com (A.R.D.); kazuyo.matsubae.a2@tohoku.ac.jp (K.M.)
* Correspondence: oita.azusa855@naro.go.jp (A.O.); adhyas@yahoo.com (T.K.A.)

1 Supplementary Figures

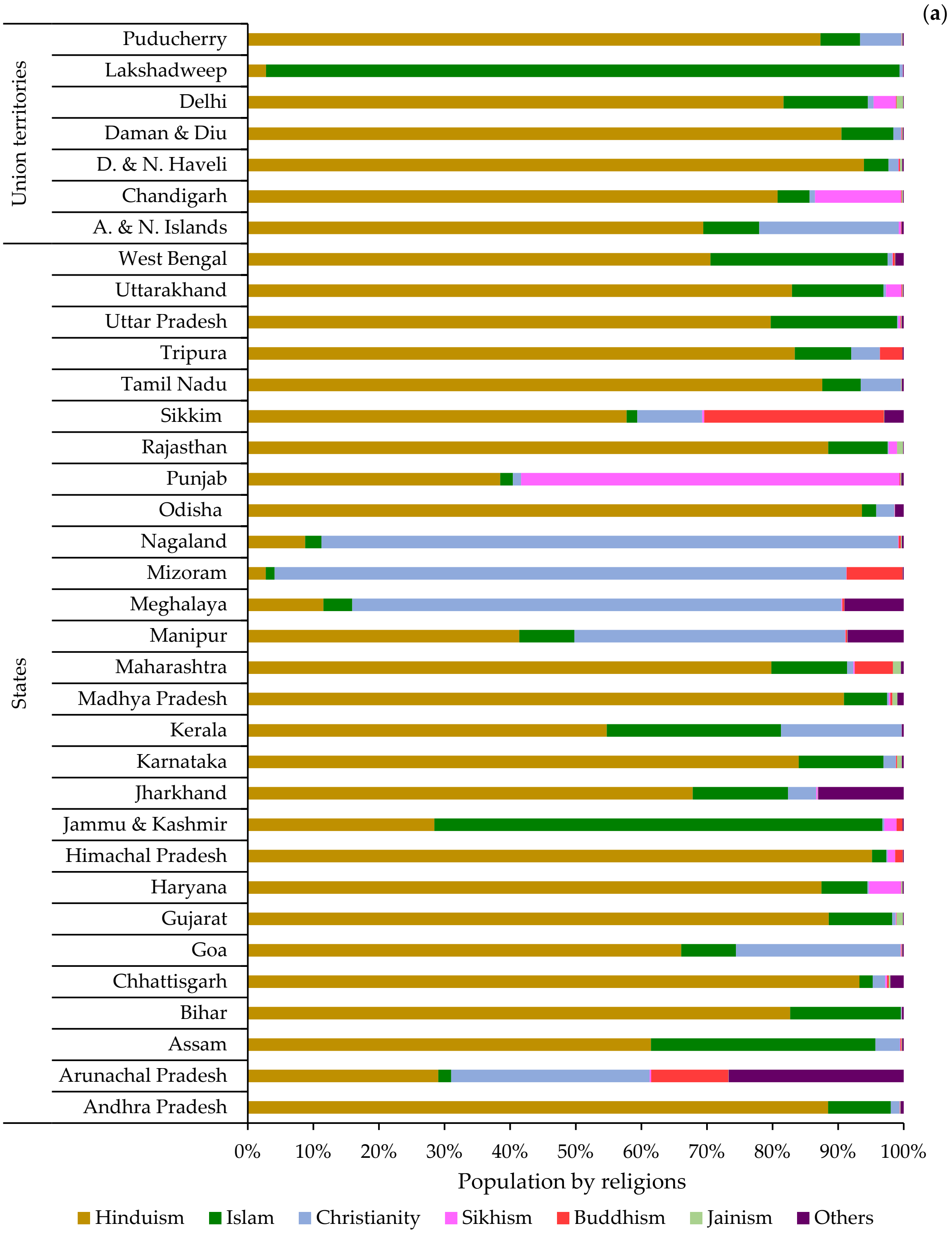

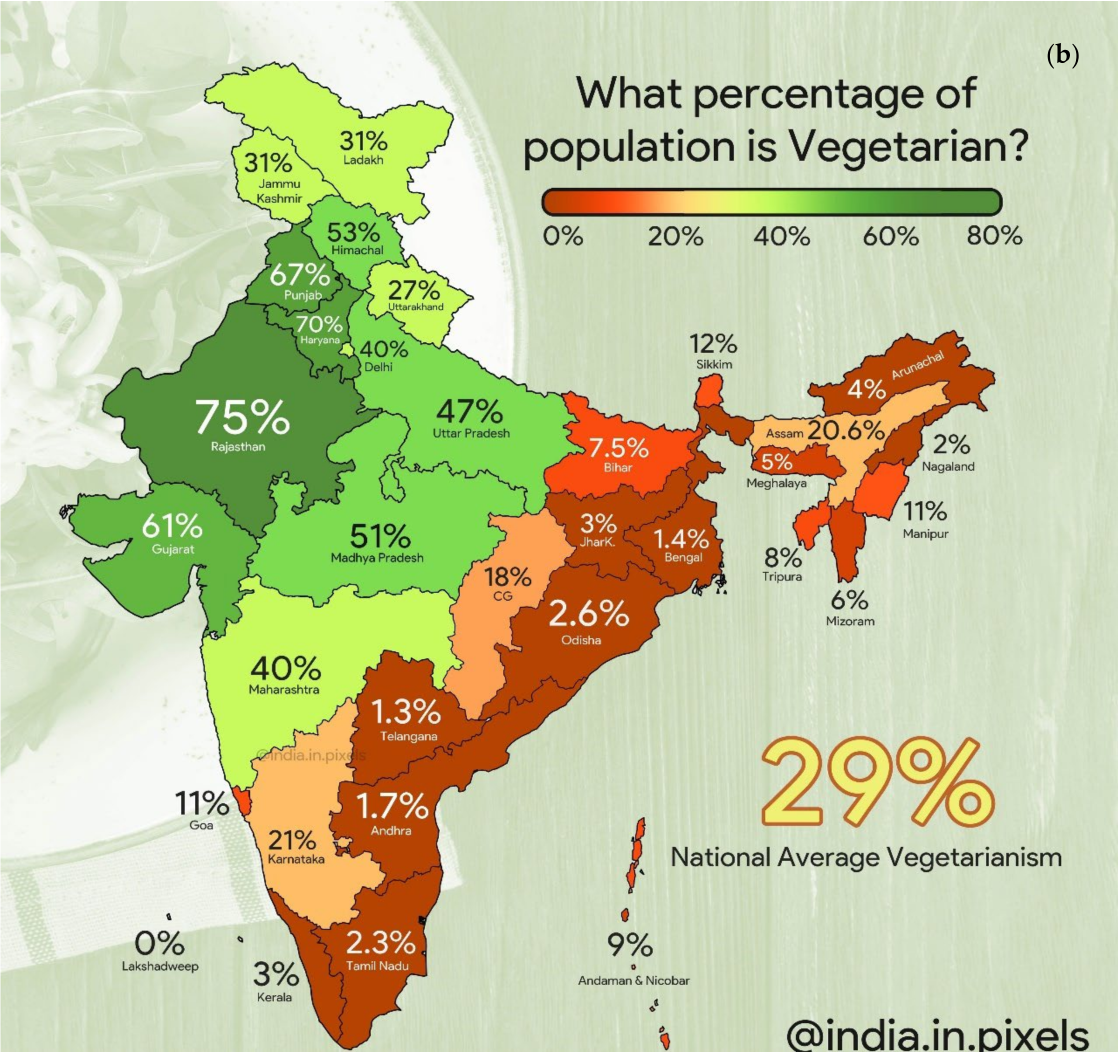

Figure S1. State and union territory-level (**a**) population by religion and (**b**) dietary choice by population in India in 2011-12.

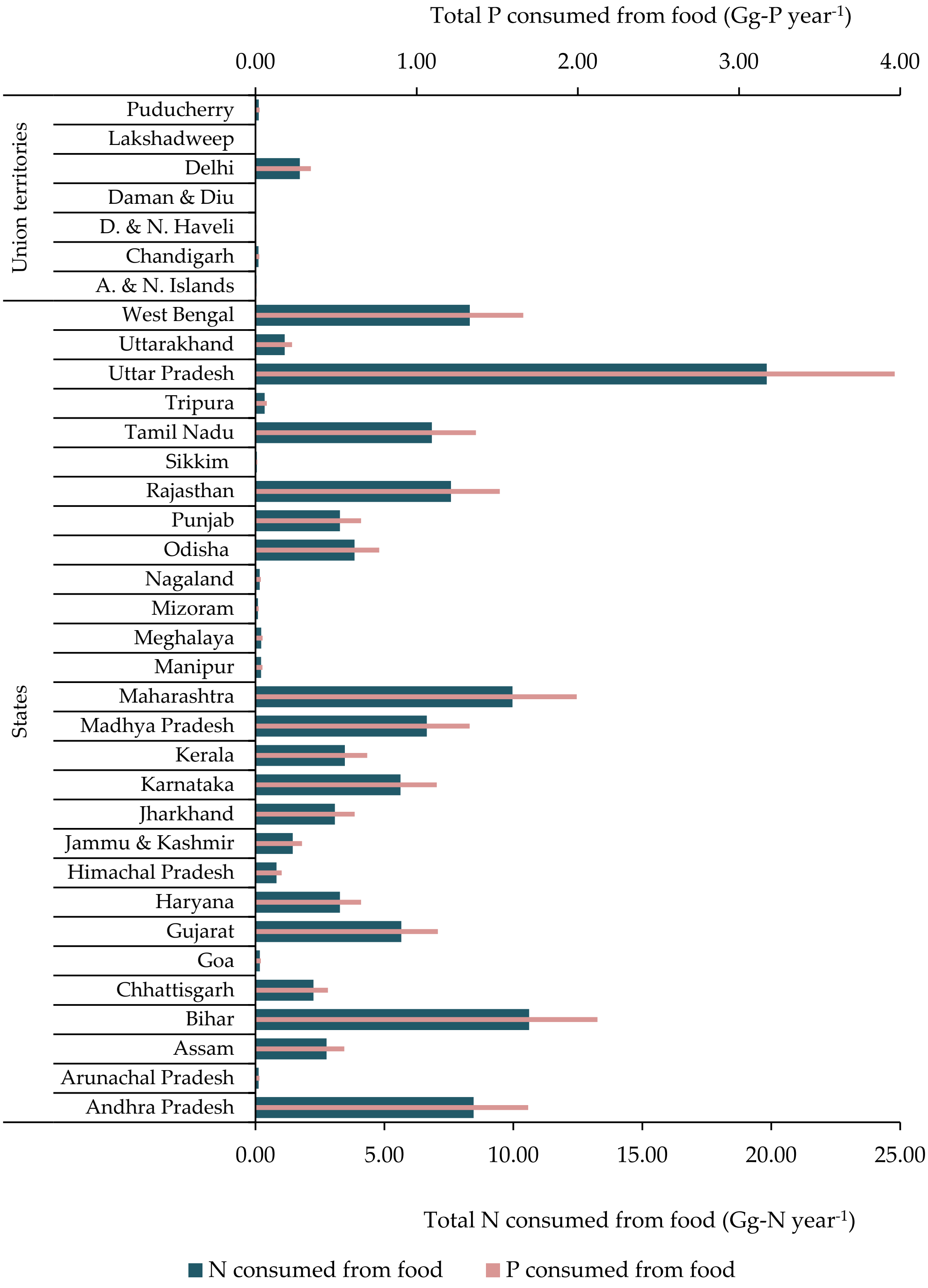

Figure S2. Total nitrogen and phosphorus consumed from food in states and union territories of India during 2011–2012.

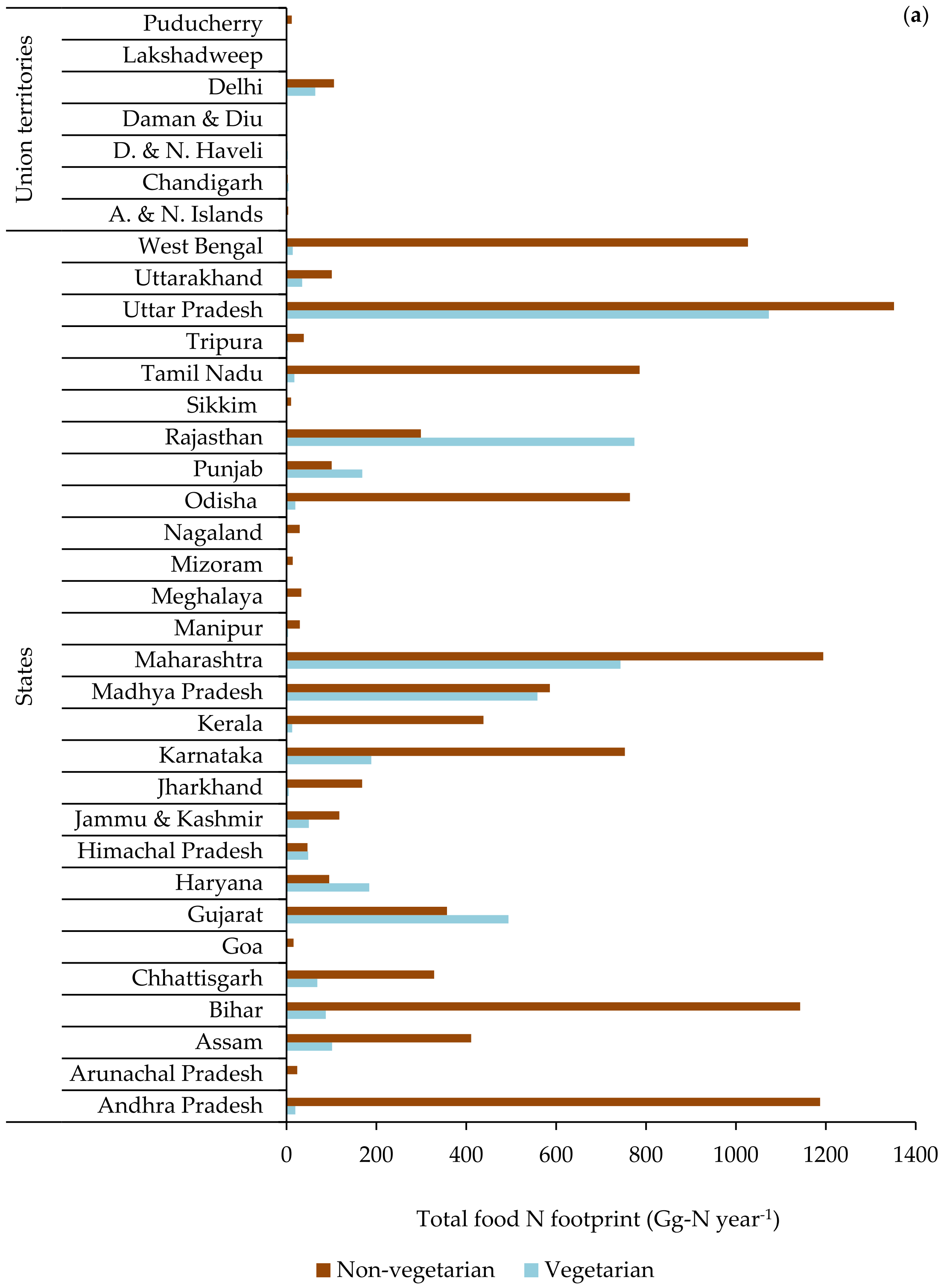
(a)
Union territories
Puducherry
Lakshadweep
Delhi
Daman & Diu
D. & N. Haveli
Chandigarh
A. & N. Islands
States
West Bengal
Uttarakhand
Uttar Pradesh
Tripura
Tamil Nadu
Sikkim
Rajasthan
Punjab
Odisha
Nagaland
Mizoram
Meghalaya
Manipur
Maharashtra
Madhya Pradesh
Kerala
Karnataka
Jharkhand
Jammu & Kashmir
Himachal Pradesh
Haryana
Gujarat
Goa
Chhattisgarh
Bihar
Assam
Arunachal Pradesh
Andhra Pradesh
0
200
400
600
800
1000
1200
1400
Total food N footprint (Gg-N year-1)
Non-vegetarian
Vegetarian

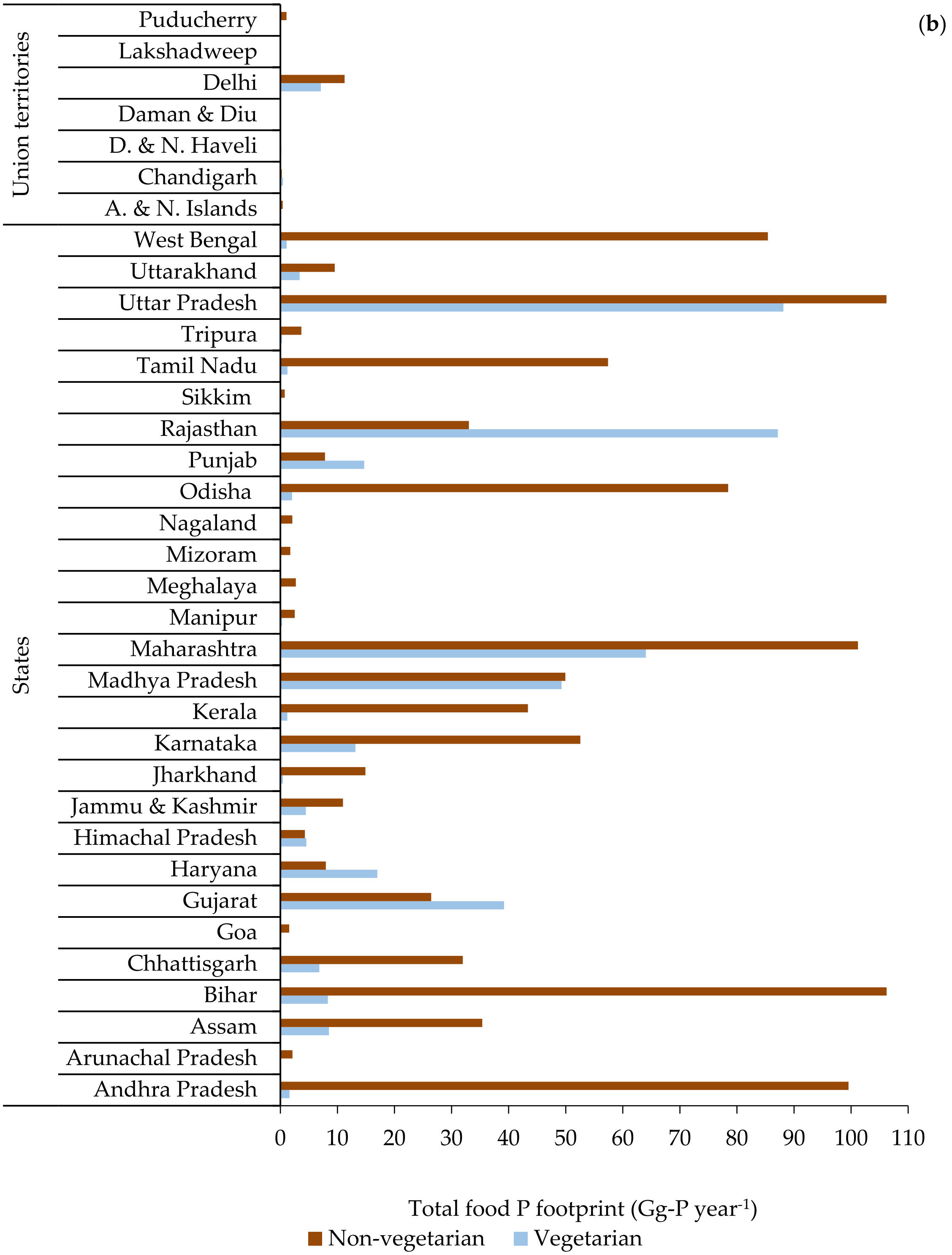

Figure S3. Total food (**a**) nitrogen and (**b**) phosphorus footprints in states and union territories of India during 2011–2012.

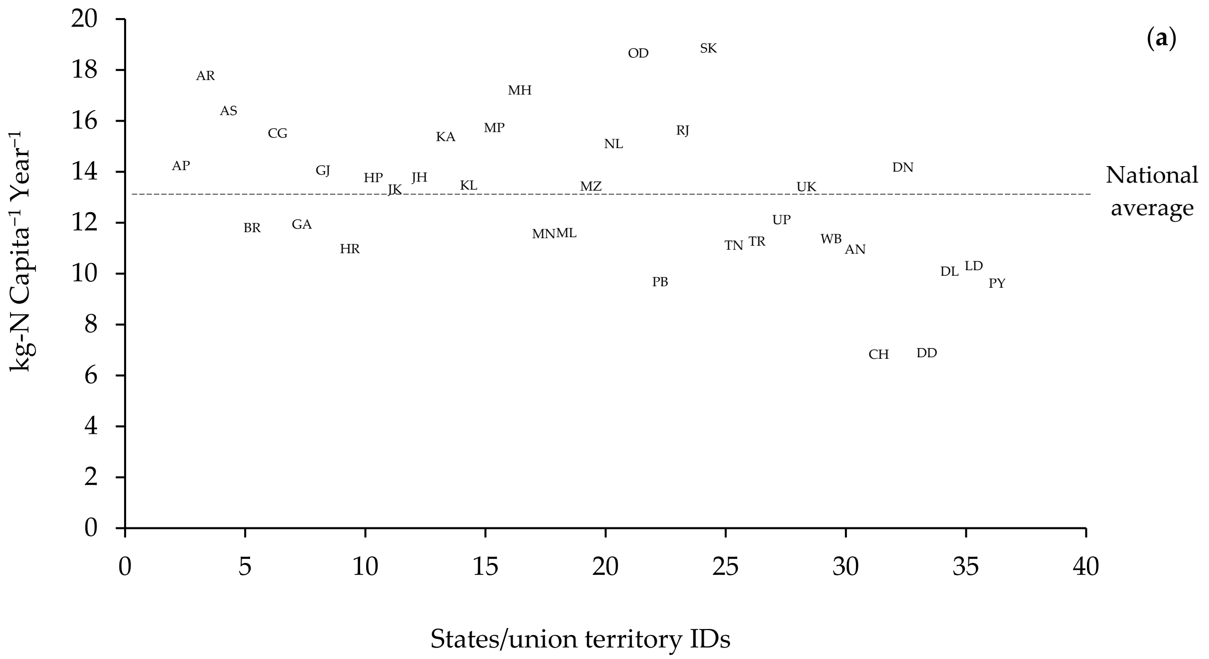

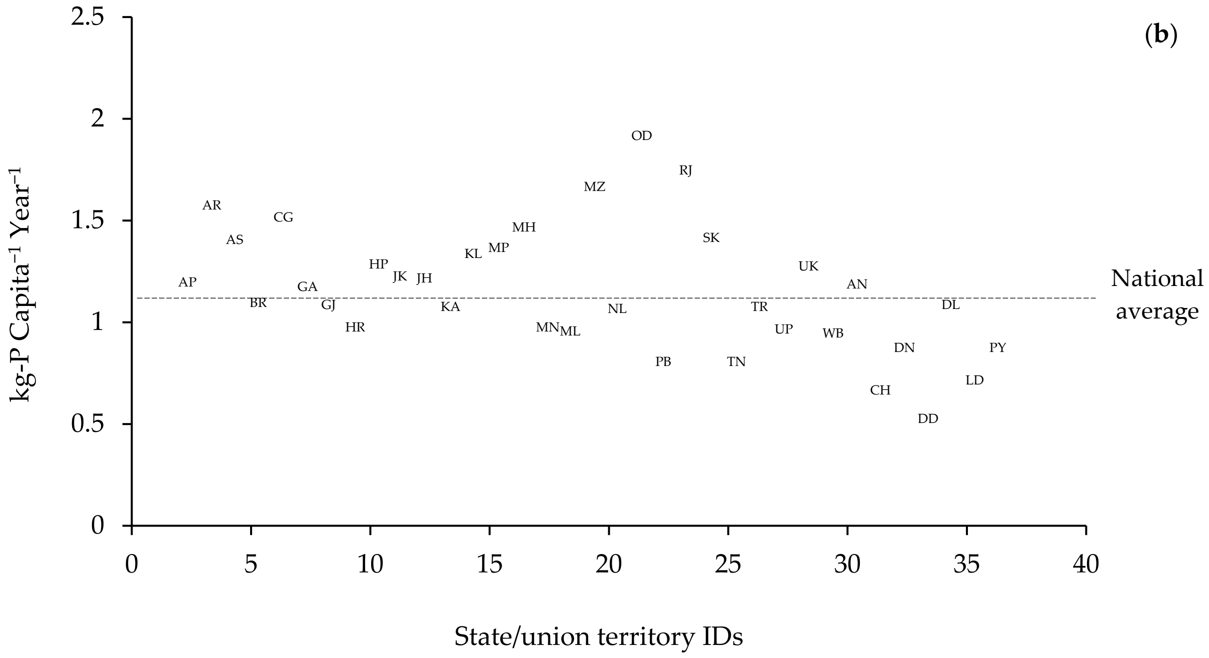

Figure S4. Differences between state and union territory level food (**a**) nitrogen and (**b**) phosphorus footprints relative to the national average during 2011–2012.

Note: State/union territory IDs are adopted from Lalwani et al. (2020) [1] in numerical order from first to last, as follows – AP: Andhra Pradesh; AR: Arunachal Pradesh; AS: Assam; BR: Bihar; CG: Chhattisgarh; GA: Goa; GJ: Gujarat; HR: Haryana; HP: Himachal Pradesh; JK: Jammu & Kashmir; JH: Jharkhand; KA: Karnataka; KL: Kerala; MP: Madhya Pradesh; MH: Maharashtra; MN: Manipur; ML: Meghalaya; MZ: Mizoram; NL: Nagaland; OD: Odisha; PB: Punjab; RJ: Rajasthan; SK: Sikkim; TN: Tamil Nadu; TR: Tripura; UP: Uttar Pradesh; UK: Uttarakhand; WB: West Bengal; AN: A. & N. Islands; CH: Chandigarh; DN: D. & N. Haveli; DD: Daman & Diu; DL: Delhi; LD: Lakshadweep; and PY: Puducherry.

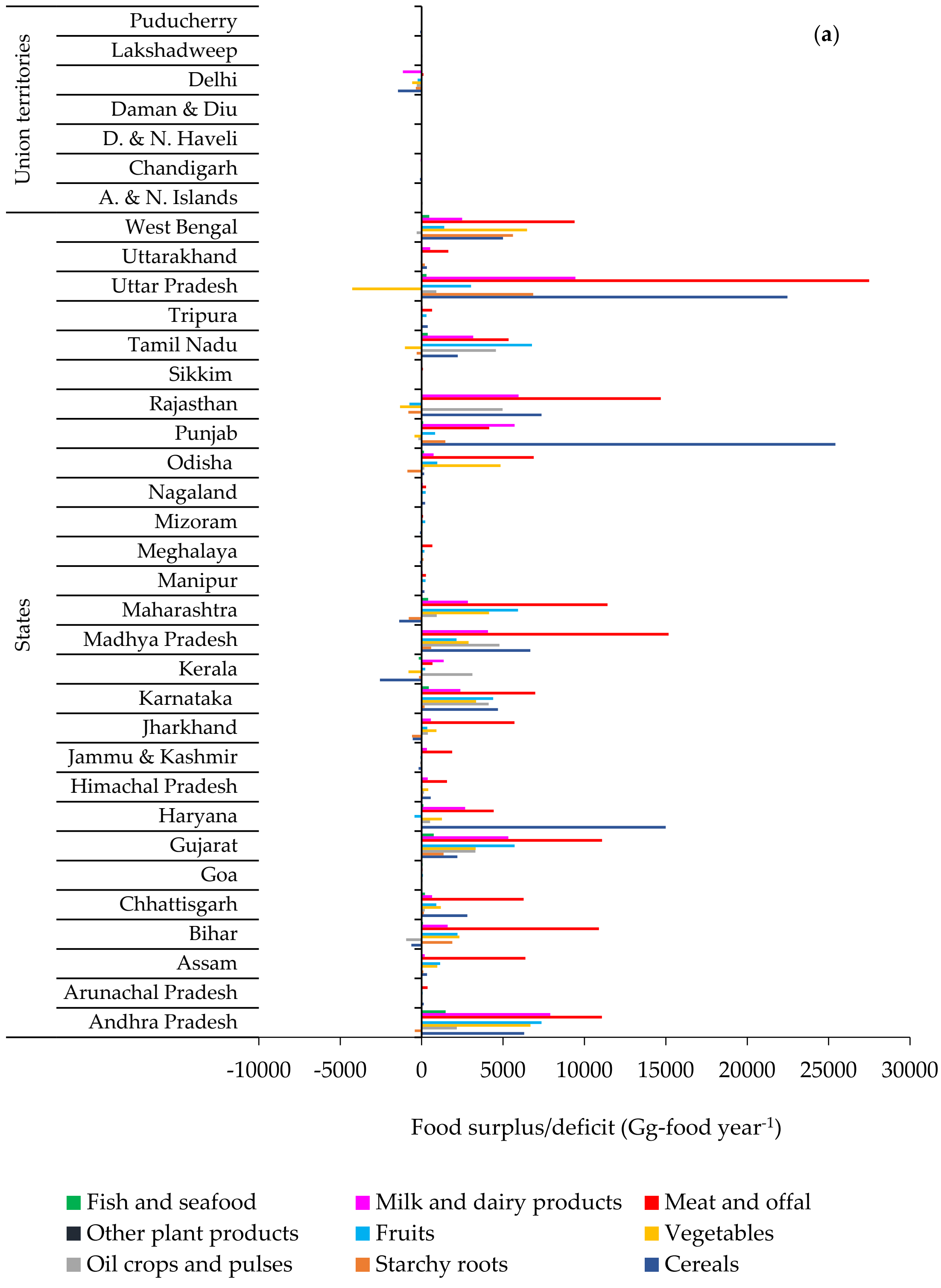
(a)
Union territories
Puducherry
Lakshadweep
Delhi
Daman & Diu
D. & N. Haveli
Chandigarh
A. & N. Islands
States
West Bengal
Uttarakhand
Uttar Pradesh
Tripura
Tamil Nadu
Sikkim
Rajasthan
Punjab
Odisha
Nagaland
Mizoram
Meghalaya
Manipur
Maharashtra
Madhya Pradesh
Kerala
Karnataka
Jharkhand
Jammu & Kashmir
Himachal Pradesh
Haryana
Gujarat
Goa
Chhattisgarh
Bihar
Assam
Arunachal Pradesh
Andhra Pradesh
-10000 -5000 0 5000 10000 15000 20000 25000 30000
Food surplus/deficit (Gg-food year-1)
Fish and seafood
Milk and dairy products
Meat and offal
Other plant products
Fruits
Vegetables
Oil crops and pulses
Starchy roots
Cereals

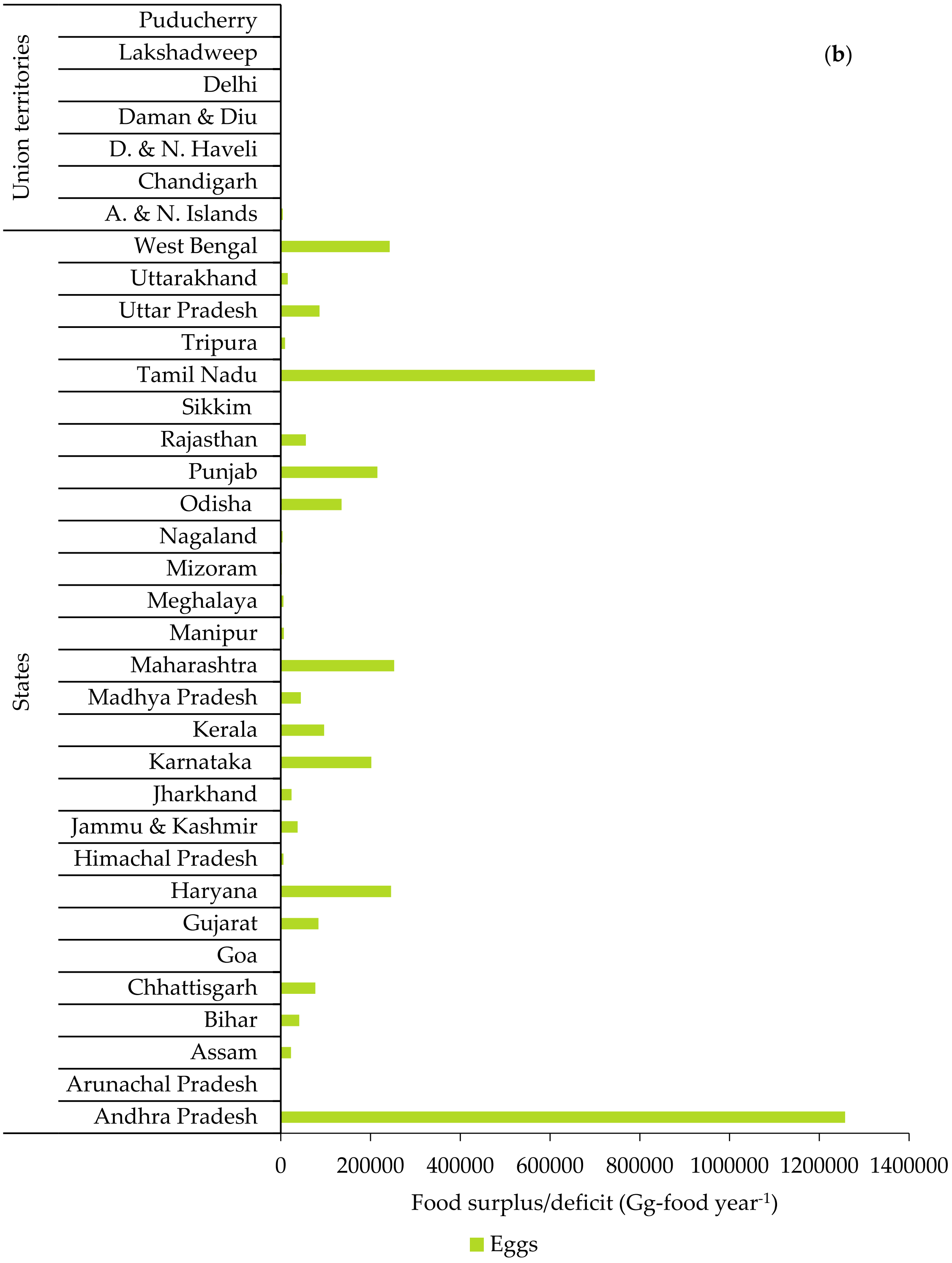

Figure S5. Surplus/deficit in (**a**) aggregated food categories and (**b**) eggs in states and union territories of India during 2011–2012.

Note: Positive values indicate food surplus and negative values indicate food deficit.
The high values for eggs relative to other food categories necessitate their presentation in a separate figure.

2 Reference

1. Lalwani, R.; Kotgirwar, S.; Athavale, S.A. Changing Medical Education Scenario: A Wakeup Call for Reforms in Anatomy Act. *BMC Medical Ethics* **2020**, *21*, 63, doi:10.1186/s12910-020-00507-0.
